# Influence of tension-band plates on the mechanical loading of the femoral growth plate during guided growth due to coronal plane deformities

**DOI:** 10.3389/fbioe.2023.1165963

**Published:** 2023-06-21

**Authors:** Lucie Hucke, Jana Holder, Stefan van Drongelen, Felix Stief, Antonio J. Gámez, Armin Huß, Andreas Wittek

**Affiliations:** ^1^ Peronalized Biomedical Engineering Laboratory, Frankfurt University of Applied Sciences, Frankfurt am Main, Germany; ^2^ Department of Mechanical Engineering and Industrial Design, School of Engineering, University of Cádiz, Cádiz, Spain; ^3^ Department of Orthopedics (Friedrichsheim), University Hospital Frankfurt, Goethe University Frankfurt, Frankfurt am Main, Germany; ^4^ Dr. Rolf M. Schwiete Research Unit for Osteoarthritis, Department of Orthopedics (Friedrichsheim), University Hospital Frankfurt, Goethe University Frankfurt, Frankfurt am Main, Germany

**Keywords:** growth plate, guided growth, finite element analysis, knee model, tension-band plate, stress distribution

## Abstract

**Introduction:** Correction of knee malalignment by guided growth using a tension-band plate is a common therapy to prevent knee osteoarthritis among other things. This approach is based on the Hueter-Volkmann law stating that the length growth of bones is inhibited by compression and stimulated by tension. How the locally varying mechanical loading of the growth plate is influenced by the implant has not yet been investigated. This study combines load cases from the gait cycle with personalized geometry in order to investigate the mechanical influence of the tension-band plates.

**Methods:** Personalized finite element models of four distal femoral epiphyses of three individuals, that had undergone guided growth, were generated. Load cases from the gait cycles and musculoskeletal modelling were simulated with and without implant. Morphological features of the growth plates were obtained from radiographs. 3D geometries were completed using non-individual Magnetic Resonance Images of age-matched individuals. Boundary conditions for the models were obtained from instrumented gait analyses.

**Results:** The stress distribution in the growth plate was heterogenous and depended on the geometry. In the insertion region, the implants locally induced static stress and reduced the cyclic loading and unloading. Both factors that reduce the growth rate. On the contralateral side of the growth plate, increased tension stress was observed, which stimulates growth.

**Discussion:** Personalized finite element models are able to estimate the changes of local static and cyclic loading of the growth plate induced by the implant. In future, this knowledge can help to better control growth modulation and avoid the return of the malalignment after the treatment. However, this requires models that are completely participant-specific in terms of load cases and 3D geometry.

## 1 Introduction

**FIGURE 1:**
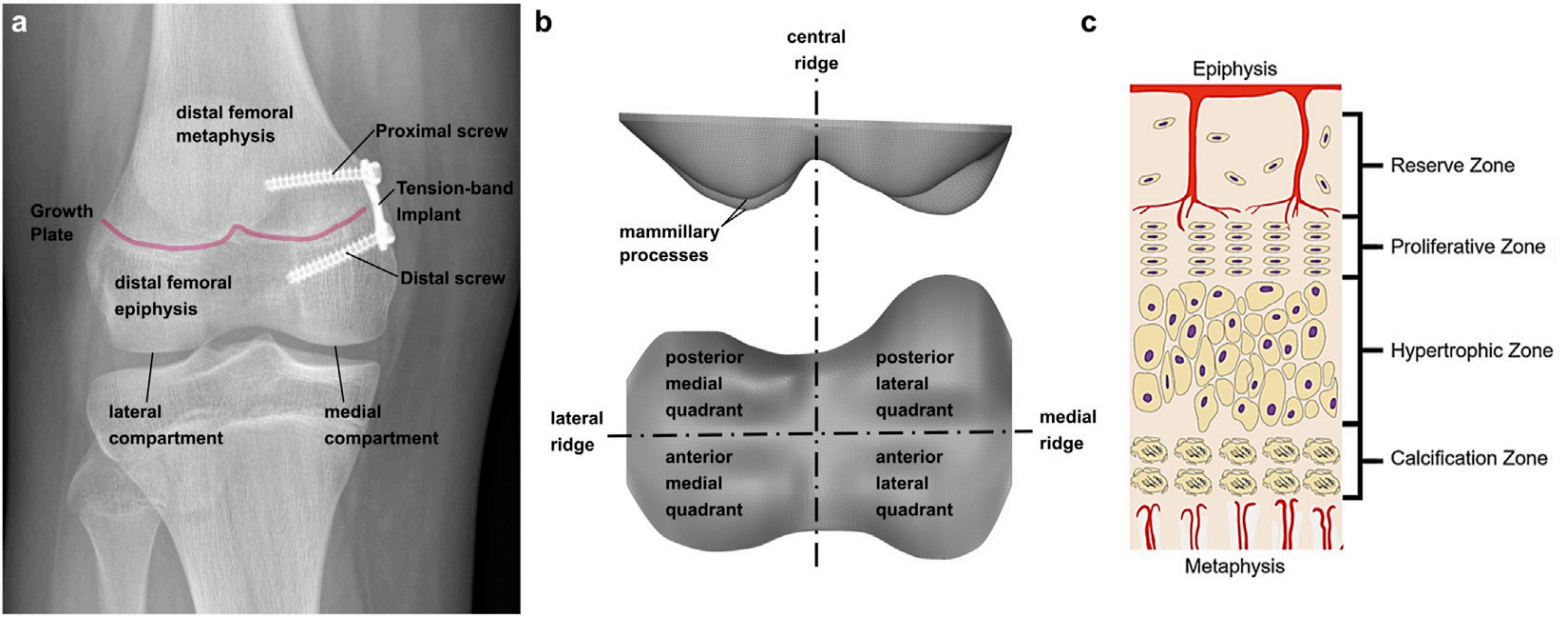
**(A)** Implant positioning at the tibio-femoral joint. Two screws, proximal and distal of the growth plate, fix the implant; **(B)** terminology of growth plate anatomy; **(C)** Growth plate zones in an epiphyseal growth plate; Reprinted from D'Andrea et al. (2021) with permission from John Wiley and Sons.

Frontal plane malalignment of the knee axis can lead to permanent unphysiological loading of the knee joint, one of the most common causes for knee osteoarthritis (Sharma et al., 2001). It can occur in one or both knees and does not have to be equally severe. A preventive therapy is the correction of the malalignment by guiding growth of bones in adolescence. Longitudinal growth of bones originates from the growth plate (GP), which changes its shape during growth until it has a complex geometry in adolescence. Presumably its shape is an adaption to the mechanical loading (Thomson, 1902; Castro-Abril et al., 2016; Stamos und Weaver, 2020; Stamos und Berthaume, 2021). The GP geometry in adolescents consists of four different compartments, the anterior medial quadrant, the anterior lateral quadrant, the posterior medial quadrant, and the posterior lateral quadrant. These are separated by a central ridge, a medial and a lateral ridge (cf. Lippiello et al., 1989; Liu et al., 2013) (Figure 1B). The GP consists of a specialized type of non-vascularized hyaline cartilage from which newly formed bone is pushed out in the direction of the diaphysis (Figure 1C) (Kronenberg, 2003) which results in a gradual transition of material properties from soft, cartilage-like tissue in the GP itself, to ossified regions (“transition zone,” cf. Sadeghian et al., 2020). It is surrounded in the transverse plane by the Ring of Lacroix (RoL), a fibrous layer that constrains the GP radially (Brighton, 1978; Piszczatowski, 2011).

The so-called “guided growth” therapy is based on the Hueter-Volkmann law. It states that static compression inhibits growth, while reduced compression can lead to accelerated growth (Gottliebsen et al., 2016; Hüter 1862a; (Huter, 1862b); Mehlman et al., 1997; Volkmann 1862). In addition Frost (1979) found that physiologically alternating compression and tension can also lead to an increase of the growth rate. Guided growth is performed by a minimal invasive surgical intervention called “temporary hemiepiphysiodesis”. The implant, a metal plate, is fixed at the GP with two screws, one in the metaphyseal and one in the epiphyseal bone (Figure 1A). This leads to a reduced growth rate on the implant side, while the normal length growth continues contralaterally, resulting in an outgrowth of the malalignment. After implant removal, normal length growth continues on both sides (Stevens, 2007; Gottliebsen et al., 2013; Gottliebsen et al., 2016). Although, the axis correction initially is successful in most of the patients, the original malalignment returns in many cases. There is still limited knowledge about this so-called rebound phenomenon and potential risk factors are not well defined and controversially reported in literature, despite a high incidence (up to 69%) (Farr et al., 2018; Stief et al., 2021b). Therefore, a deeper understanding of the growth modulation mechanism is necessary, to better understand this rebound phenomenon. To date, the success of the guided growth treatment depends on the existing residual growth and the right timing of the removal of the implant. To determine the optimal time point, there are different approaches. The clinical standard is the malalignment test, where the mechanical leg axis and the deviation of the center of the knee joint from said axis is measured on radiograph images in the frontal plane (Vogt et al., 2014). Especially in borderline cases, when the malalignment test does not reveal a clear medical indication for or against the guided growth intervention, knee joint moments from instrumented gait analysis are additionally assessed. (Böhm et al., 2015; Stief et al., 2021a). In particular, the knee adduction moment is a commonly used surrogate measure for medial and lateral compartment knee loading. It was statistically associated to osteoarthritis severity and progression. A valgus malalignment was shown to reduce the knee adduction moment and therefore at the time of removal of the implant it should no longer differ from an age-matched control group (Holder et al., 2020). Calculating knee joint contact forces requires the additional use of musculoskeletal simulation software. While calculation of joint reaction forces using inverse dynamics represents the reaction to external loads, only musculoskeletal modelling enables to additionally consider the contribution of the internal loads, mainly generated by muscles during walking (Lerner et al., 2015; Rajagopal et al., 2016), and are more representative of cartilage loading (Dell'Isola et al., 2017). Holder et al. (2020) used the data obtained by instrumented gait analysis to generate participant-individual multi-body models in OpenSim und calculated the medial and lateral knee contact forces (KCF), considering the influence of the muscle forces.

The described approaches consider the global mechanical loading of the whole knee joint in the form of resulting moments and forces. They do not investigate how these global loads on the knee translate into a load on the soft tissue in the growth plate, from which growth originates. Due to the complex geometry of the distal femoral growth plate, a balanced ‘global’ load does not necessarily mean a balanced or uniform local load on the tissue. This local loading of the growth plate tissue is exactly where the implant intervenes, since the joint momentum and the medial and lateral knee contact forces do not change with implantation. The finite element (FE) method that was used in this study is appropriate to analyze locally varying mechanical loading of tissue regions with complex, irregular geometries and its dependency on external loads and boundary conditions. To our knowledge the current study is the first that applies the FE method to investigate how tension-band plates change the distribution, type and size of mechanical stresses in the juvenile growth plate. This knowledge is a necessary precondition for analyzing and modelling differences of length growth of bones as a response to changes in mechanical loading.

FE models of the distal femoral GP are rare in literature. Most studies assume a simplified geometry. Mainly, linear elastic material properties are assumed, which show a wide range of the Young´s Modulus from 0.2 up to 1,157 MPa with an accumulation around 6 MPa. The Poisson-Ratio varies between 0.1 and 0.5 but is mostly assumed to be 0.48. (Piszczatowski, 2011; Yadav et al., 2017; Sadeghian et al., 2021; Stamos und Berthaume, 2021).

Schneider et al. (2018) inversely estimated the forces exerted by tension-band plates from the bending of the screws. The model focused on the material and geometrical properties of the screws and used a simplified cubic geometrical model of the trabecular bone with an embedded plane GP. Gao et al. (2017) used two cubic models of the trabecular bone with a “flat” and an “m-shaped” GP. The focus of this model was to investigate how the “global” forces are affecting the local cells at different depths of the tissue. Using geometrically simplified mechanical models with different morphological parameters, several studies have shown that GP morphology is an important factor influencing the transfer of joint loading to the stress distribution in the GP tissue itself (Piszczatowski, 2012; Castro-Abril et al., 2015; Guevara et al., 2015; Stamos und Berthaume, 2021). Stamos und Berthaume (2021) investigated the relations between GP morphology, type of biomechanical loading and resulting GP stresses in a comparative study between human and chimpanzee motion patterns. In order to predict the effect of mechanical loading on bone growth, several FE studies (cf. Carriero et al., 2011; Piszczatowski, 2012; Yadav et al., 2017; Sadeghian et al., 2021) apply the Osteogenic Index (OI that was proposed by Carter und Wong (1988); Stevens et al. (1999)). The OI is a linear combination of the maximum octahedral shear stress (stimulation) and the minimum hydrostatic stress (inhibition) adjusted by experimental data. It was developed to predict the endochondral growth and ossification in long bones in children from 8 weeks to approximately 2 years after birth. Since this model is not validated–though often used–for other age groups and, moreover, several, mutually contradictory versions of the OI have been proposed since the first use, the authors of this study decided not to use the OI or similar indices. This is discussed in more detail in the discussion section.

Although it is known that the mechanical loading situation at the knee joint during gait can affect the development of the leg axis (Stokes et al., 2002), the influence of guided growth on the mechanical loading of the GP tissue has not yet been studied using personalized FE analyses, and thus a geometrical complex GP. Furthermore, a differentiation between static and cyclic compression was not considered in previous studies. Therefore, the objective of this study was to analyze the influence of tension-band plates on the mechanical loading on the GP in terms of locally varying stress distributions in the plate itself. For this purpose, FE models of patients undergoing frontal plane leg axis correction by guided growth were generated at different stages of the treatment. Characteristic load cases were obtained from the results of instrumented gait analyses and multi-body simulations that had been performed for a previous study by Holder et al. (2020). Characteristic geometrical features of the individual GP at different stages of the treatment were derived from radiographs and used to generate 3D models with adequate morphological complexity even though available data did not allow for fully participant-individual 3D models. To the best of our knowledge, this is the first time the FE method was used to assess the loading of the GP due to the implant. Our hypothesis is, that the implant reduces the growth rate by increasing the static compression and reducing the cyclic changes of dynamic loading during gait on the implant side.

## 2 Materials and methods

### 2.1 Participants

In the present study, three patients with a pathological valgus alignment of at least one knee and a clinical indication for a temporary hemiepiphysiodesis were included (Table 1). Clinical indications for the in- and explantation of the implant were based on static weight bearing full-length radiographs of the legs in the frontal plane. Additionally, an instrumented gait analysis was performed a few days before implantation and explantation. Participants and their parents gave written informed consent to participate in this study, as approved by the local ethics committee (182/16) and in accordance with the Declaration of Helsinki.

**TABLE 1:** Study participant’s characteristics. The static mechanical axis angle (MAA) is the angle between the mechanical femur axis and the mechanical tibia axis in the frontal plane. Negative values of MAA indicate a valgus malalignment, positive values a varus malalignment. Table includes the thickness of the growth plate preoperative and postoperative: at the beginning of the treatment, a constant thickness was assumed, at the end of the treatment (explantation of the implant), the thickness was measured on the implant side and on the contralateral side.

Patient	P1		P2	P3
Sex	Female		Male	Male
Analyzed leg	Left	Right	Right	Right
Preoperative				
Age in years	11		13	14
Height in cm	162.5		169.3	175.0
Body weight in kg	74.2		71.0	60.8
MAA in °	−5	−3	−6	−10
Thickness growth plate in mm	1.43	1.35	1.51	1.39
Postoperative				
Age in years	13		15	15
Height in cm	167.8		178.5	180.0
Body weight in kg	91.3		88.5	70.9
MAA in °	−3	1	3	−2
Thickness growth plate in mm implant side/opposite side	0.47/0.66	0.46/0.72	0.69/1.20	0.5/0.76

### 2.2 Gait analysis and musculoskeletal modelling

In order to assess the dynamic mechanical loading on the GP the results of instrumented gait analysis and multi-body simulations from a previous study by Holder et al. (2020) were used as boundary conditions for the FE analyses. The gait analysis and musculoskeletal modelling was performed before and after guided growth as described in detail in Holder et al. (2020). Kinematic data were collected using an 8-camera motion capture system (MX 10, VICON Motion Systems, Oxford, UK) while participants walked barefoot. Ground reaction forces were recorded synchronously using two force plates (Advanced Mechanical Technology, Inc., Watertown, MA, United States) situated at the mid-point of the walkway. A custom-made lower body protocol was used for improvement of the reliability and accuracy described in a previous study Stief et al. (2013). In addition to the standardized Plug in-Gait marker set (Kadaba et al., 1990), reflective markers were attached on the medial malleolus, medial femoral condyle and greater trochanter. The statically measured midpoints between the medial and lateral malleolus and condyle markers defined the centers of rotation of the ankle and knee joints (Stief et al., 2013). The center of the hip joint was calculated with a standardized geometrical prediction method using regression equation (Davis et al., 1991) which is common in the clinical gait community (Stief, 2018). Three to five dynamic trials with a clear foot-force plate contact were selected for further processing.

For subsequent generation of a musculoskeletal model in OpenSim (version 3.3), marker and force plate data were processed using the MOtoNMS toolbox (version 3) in Matlab (version 2022a, The MathWorks, Inc., Natick, MA, United States). The full body model by Lerner et al. (2015) was used. This model includes 18 body segments and 92 muscle-tendon actuators and allows for the separate calculation of medial and lateral KCFs. The model was linearly scaled based on the marker positions to fit the participant’s body mass and height. The participant-specific mechanical axis angle, as obtained from full-length standing anteroposterior radiograph was implemented. Inverse kinematics and inverse dynamics, were calculated within OpenSim for all participants. A static optimization implementation that incorporates tendon compliance and passive muscle forces was used to solve for muscle activations, with a cost function that minimized the sum of squared muscle activation (Seagers et al., 2022). The computed medial and lateral KCFs were time normalized to the full duration of the stance phase, beginning with the foot strike and ending with the foot off (Holder et al., 2020).

### 2.3 Finite element model

#### 2.3.1 3D geometries and FE discretization

**FIGURE 2:**
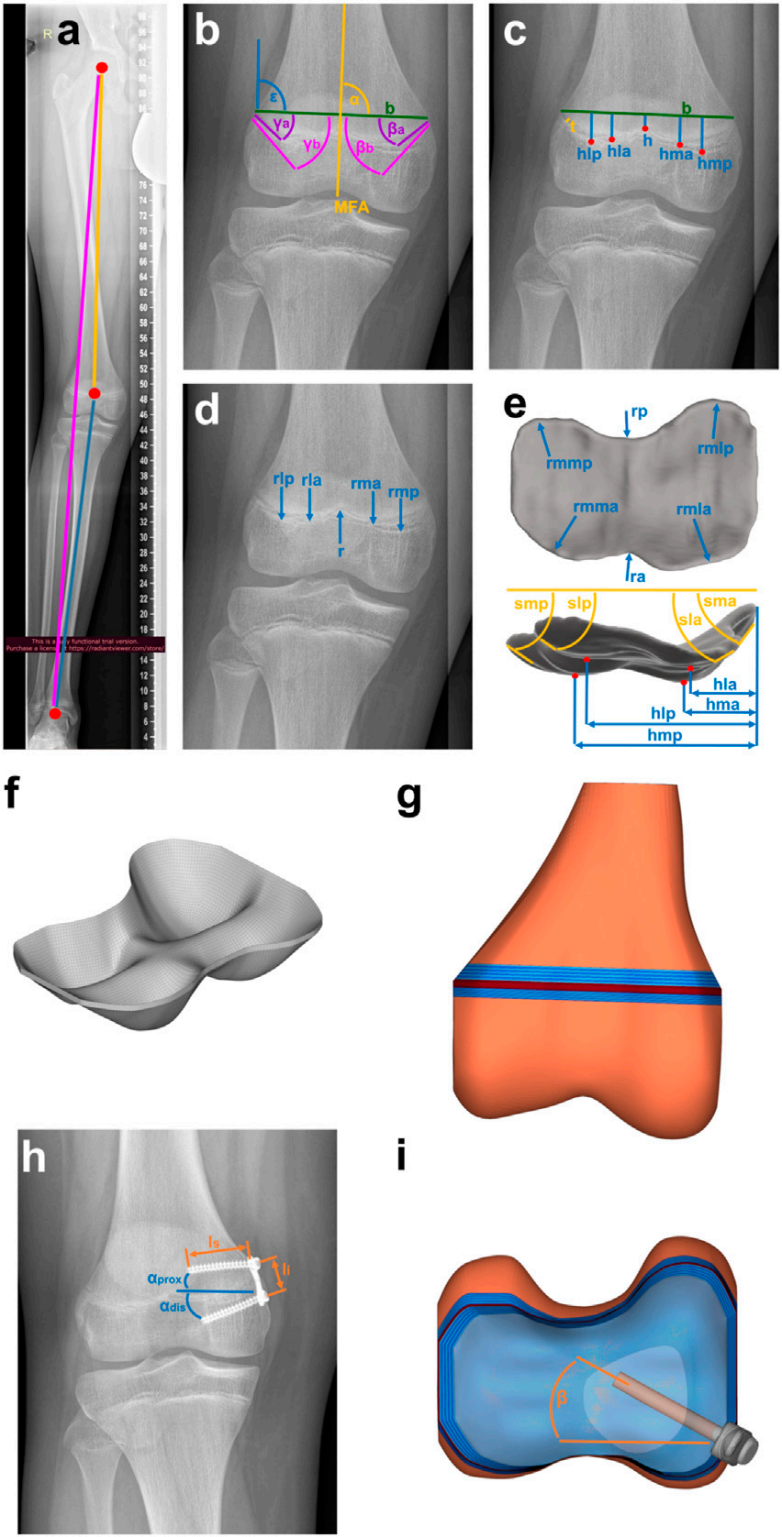
Creating the geometry based on clinical data: **(A–E)** Characteristic parameters of the growth plate and the knee, such as **(A)** malalignment, **(B)** edge steepness measured with angles medially β′ and laterally,γ′ each anterior,a’ and posterior, p’, **(C)** location of vertices, h’ laterally, l’ and medially, m’ each anterior, a’ and posterior, p’, width, b’ and thickness, t’, **(D)** radii, r’ laterally, l’ and medially, m’ each anterior, a’ and posterior, p’ and **(E)** additional parameters from 3D-MRI-data (Lustig und Vasanawala, 2022) such as the radii in the transversal plane, rm’, r’ laterally, l’ and medially, m’ each anterior, a’ and posterior, p’. This leads to **(F)** the 3D-Mesh of growth plate, implemented in **(G)** FE-Mesh of distal femur. **(H)** Implant parameters such as screw length, l_S’_, implant length, li’ and screw angles,α_prox_’, α_dis_’ are measured and implemented in **(G)** FE-Mesh of the distal femur with **(I)** the implant included.

To create a geometrical three-dimensional (3D) model of the distal femoral GP of each participant, characteristic parameters were measured manually in radiographs using Fusion 360 (Fusion 360, Autodesk, San Rafael, United States): width and thickness, axis malalignment, edge steepness, location of vertices of valleys and ridges, radii at the vertices, and implant positioning in the frontal plane. To determine the thickness the shortest distance between the proximal and distal border of the growth plate was measured at a location where it was clearly visible. For the axis malalignment, the mechanical leg axis connecting the ankle joint and the hip joint was defined and the deviation of the knee joint center from the axis was measured (Figure 2A). For reference a line connecting the outwards, upper points of the GP was introduced (Line b, Figure 2B), the length of this line was considered the width. The edge steepness was measured by the angle of a straight tangential line at the edge of the GP and the Line b (Figure 2B). The radii (Figure 2D) and location of vertices of valleys and ridges (Figure 2C) were defined by placing points at the locations and fitting a circle on the local shape of vertices and ridges. Vertices and steepness were measured for the anterior and posterior part of the GP separately, based on the assumption that the distal structures lie posterior. The implant positioning in the frontal plane (Figures 2H, I) was measured by the angles between the screws and a vertical line. Additionally, the screw length and the implant size were measured. To minimize the random error of manual measurement, a total of five measurements for each radiographic image were performed in a random order of patients and by the same person and the mean value was used. To complete the 2D radiograph image data into a 3D geometry, radii and edge steepness in the transversal plane were measured on open source Magnetic Resonance Images (MRI) data (Lustig und Vasanawala, 2022) (Figure 2E).

A cloud with a total of 59 points was generated from the measured parameters. These points were interpolated in two steps using a shape preserving piecewise cubic interpolation (Matlab function PCHIP). Using Hypermesh Desktop (HyperMesh 2017; Altair HyperWorks, Troy, Michigan, United States), the resulting surface was discretized into quad elements with an edge length of 0.75 mm. This 2D mesh was dragged to the measured thickness of each GP. A 3D mesh of hexaeder and wedge elements (C3D6, C3D8), which was organized in 5 to 6 layers over the thickness (Figure 2F), resulted. A convergence study was performed to determine the optimal element size, as shown in the Supplementary Material (Section 1).

**FIGURE 3:**
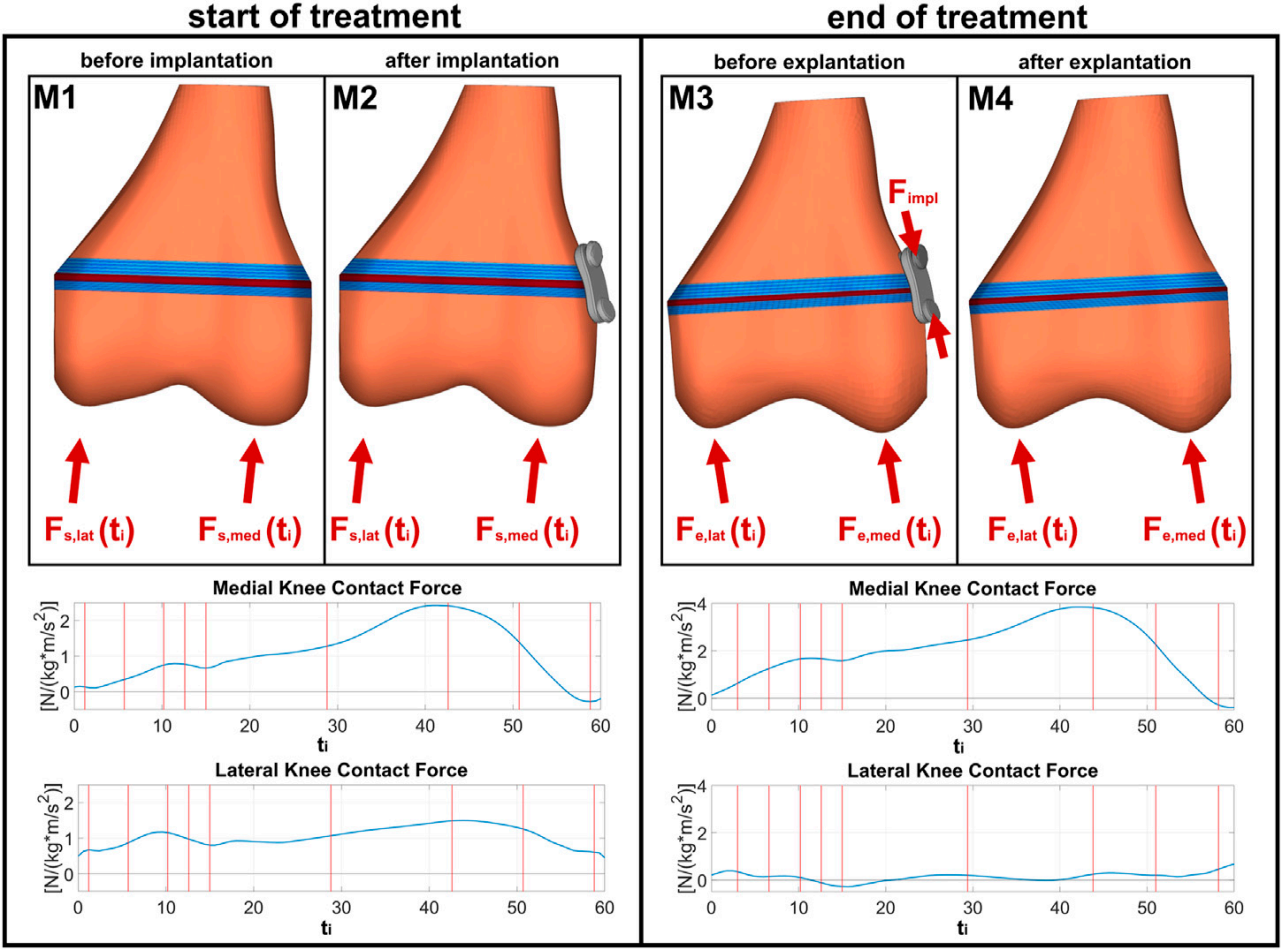
FE models for different stages of treatment: M1 and M2 are models before and after implantation at the start of the treatment, with the same boundary conditions obtained from corresponding individual instrumented gait analyses; M3 and M4 are models before and after explantation at the end of the treatment, with now changed boundary conditions due to growth and correction of malalignment.

The computed medial and lateral KCFs were concentrated forces that pass through the joint axis of the knee. Since the GP consists of a very soft material, it was not possible to apply the forces directly to it, this would have led to infinitely high stresses at one point (singularity). Therefore, the GPs were embedded in an idealized distal femur geometry that allowed the application of the medial and lateral KCFs onto the bone in sufficient distance to transform concentrated loads into realistically distributed stresses acting on the GP. Femoral bone geometry was the same for all participants. The idealized distal femur geometry consisted of the medial and lateral joint compartments, the distal femoral trabecular bone, distal and proximal transition zones between GP, bone, and the RoL (Figure 2G). The RoL is a fibrous tissue that lies circumferentially around the GP. Without the RoL, the GP would be pressed out between the epiphyseal and metaphyseal femur and thus change its shape at the edge. This would cause notch effects, i.e., singularities, in the peripheral region of the GP. Discontinuities in stiffness also lead to stress artefacts in the peripheral regions, which were prevented by considering the transition zones (Sadeghian et al., 2020) in the model. The effects of the different components on the stress distribution are shown in the Supplementary Material, Section 2 and Supplementary Figures S6, S7. As the thickness of the distal and proximal transition zones, 3 and 5 mm were chosen (Sadeghian et al., 2020; Castro-Abril et al., 2015; Figure 2G). The RoL was modelled by 4-node shell elements (S4). The implant and the screws were discretized by 8-node brick elements and 4-node tetrahedron elements (C3D4/C3D8). All used element types are linear and of full integration type. For the execution of the simulations the Solver Abaqus 6.14 (Dassault Systèmes Simulia Corp., Vélizy-Villacoublay, France) was used (Table 2). The angle of the screws in the transversal plane was calculated by comparing the length of the projection of the screws on the frontal plane measured from the radiographs and the known true length of the screws reported by the surgeon (Figures 2H, I). All GP parameters were measured at two time points, before implantation and before explantation. From these measurements four different FE-models, M1 to M4, of each knee were created, representing different stages of treatment: M1 and M2 model the knee at the start of treatment before and after implantation of the tension-band plate. M3 and M4 refer to the end of treatment before and after explantation (Figure 3). For all components, linear elastic material properties with Young´s Modulus of 6 MPa for the GP, 2,942 MPa for the trabecular bone, 775 MPa for the RoL and 110,000 MPa for the implant and the screws were used. The transition zone has a stepwise increasing Young´s Modulus from GP to bone. The Poisson-Ratio was 0.48 for the GP and 0.3 for the other components (Table 2).

**TABLE 2:** Mesh and material properties of FE-model. With two given element types, underlined element types are the mainly used ones for each component. The used solver was Abaqus 6.14. Material properties are linear elastic.

Part	Element type	E-Modulus [MPa]	Poisson-ratio	Source
Growth Plate	C3D6/C3D8	6	0.48	Yadav et al., 2017
Transition Zone	C3D6/C3D8	6–2,942	0.3	-
Trabecular Bone	C3D4/C3D8	2,942	0.3	Yadav et al., 2017
Perichondrial Ring	S4	775	0.3	Fishkin et al., 2006
Implant	C3D4/C3D8	110,000	0.3	-
Screws	C3D4/C3D8	110,000	0.3	-

#### 2.3.2 Load cases und boundary conditions

In the following, the terminus “load case” stands for the different loads at different points of the gait cycle, whereas the terminus “stage” stands for the different stages of treatment: start and end of treatment. For each of the four FE-models stress distributions in the GP were evaluated for nine different characteristic load cases throughout the stance phase of the gait cycle (cf. e.g., Perry und Burnfield, 2010): initial contact, end of loading response, mid stance, terminal stance (maximum KCF), pre-swing, and four intermediate cases in the middle between (Figure 3). For each load case, medial and lateral KCF vectors obtained from the joint reaction analysis were applied as quasistatic loads. The only difference between models M1 and M2 as well as between M3 and M4 was the existence of the implant. In M2 the implant is stress free, whereas in M3 the implant is under pre-tension stresses due to the bone growth. Schneider et al. (2018) have reported implant forces ranging from 129 to 1002 N. For the present study, the minimum force (129 N), mean of all the reported forces (485.1 N), and the maximum force (1002 N) were chosen for all participants. In order to apply this to the models, the augmented iterative design algorithm by Rausch et al. (2017) was used.

An additional study was conducted, in which one load case (terminal stance of P2) was applied to the models M1 of the patients P1 R, P2 R and P3 R. P1 L was excluded since it was a left knee. The aim of this study was to show the influence of the GP geometry on the stress distribution in the GP under the same boundary conditions.

### 2.4 Stress metrics

To account for the different influences of compression, tension and shear, the hydrostatic and octahedral shear stress distributions in the GP were calculated from the Cauchy stress tensor using an in-house Matlab script.

The hydrostatic stress was calculated as

$$\sigma_{Hi}=-\frac{1}{3}tr\left(\sigma_{i}\right)$$ (1)

with $\sigma_{i}$ being the stress tensor. And the octahedral shear stress as

$$\sigma_{Si}=\sqrt{-\frac{1}{3}\left[{tr}^{2}\left({\sigma_{i}}^{Dev}\right)-tr\left({\sigma_{i}}^{{Dev}^{2}}\right)\right]}$$ (2)

with ${\sigma_{i}}^{Dev}$ being the deviatoric stress tensor of $\sigma_{i}$ :

$${\sigma_{i}}^{Dev}=\sigma_{i}-\frac{1}{3}tr\left(\sigma_{i}\right)I.$$ (3)

I is the Identity Tensor.

**FIGURE 4:**
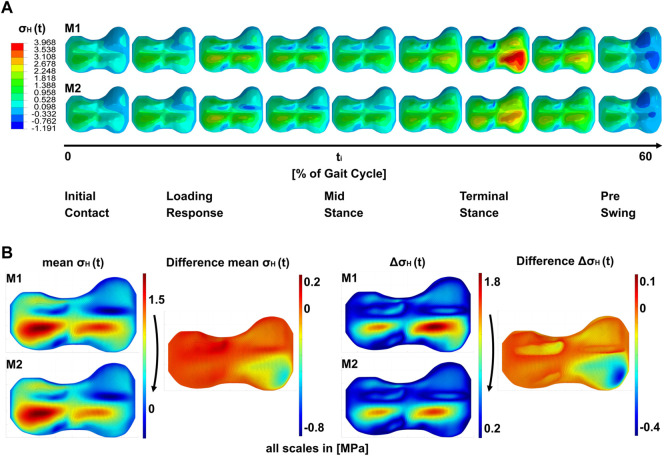
Results of the simulations; **(A)** shows a timeline of hydrostatic stresses in the growth plate during the stance phase of the gait cycle for models M1 and M2. i.e., both models are identical with regard to load cases and geometry, but differ in the absence or presence of the implant (M1 and M2, respectively). **(B)** shows the peak-to-peak amplitude Δσ_H_ (t) over the gait cycle and the mean σ_H_ (t) over the gait cycle. Both shown for M1 and M2. Right to this the difference plots between M1 and M2 are shown.

The results of the simulations (Figure 4) are the stress distributions in the GP for each load case throughout the gait cycle examined at four stages of the treatment (M1–M4, Figure 4A). The peak-to-peak-amplitudes and the mean of the stress metrics $\sigma_{Si}$ and $\sigma_{Hi}$ were calculated over the gait cycle for each model and each element. The peak-to-peak-amplitudes show the cyclic change of the loading of the growth plate during gait whereas the mean represents the static loading of the growth plate. For a better visualization of the impact of the implant, the difference of each value from before and after implantation at the start of treatment (M1 to M2) (Figure 4B) and from before and after explantation (M3 to M4) at the end was calculated.

## 3 Results

**FIGURE 5:**
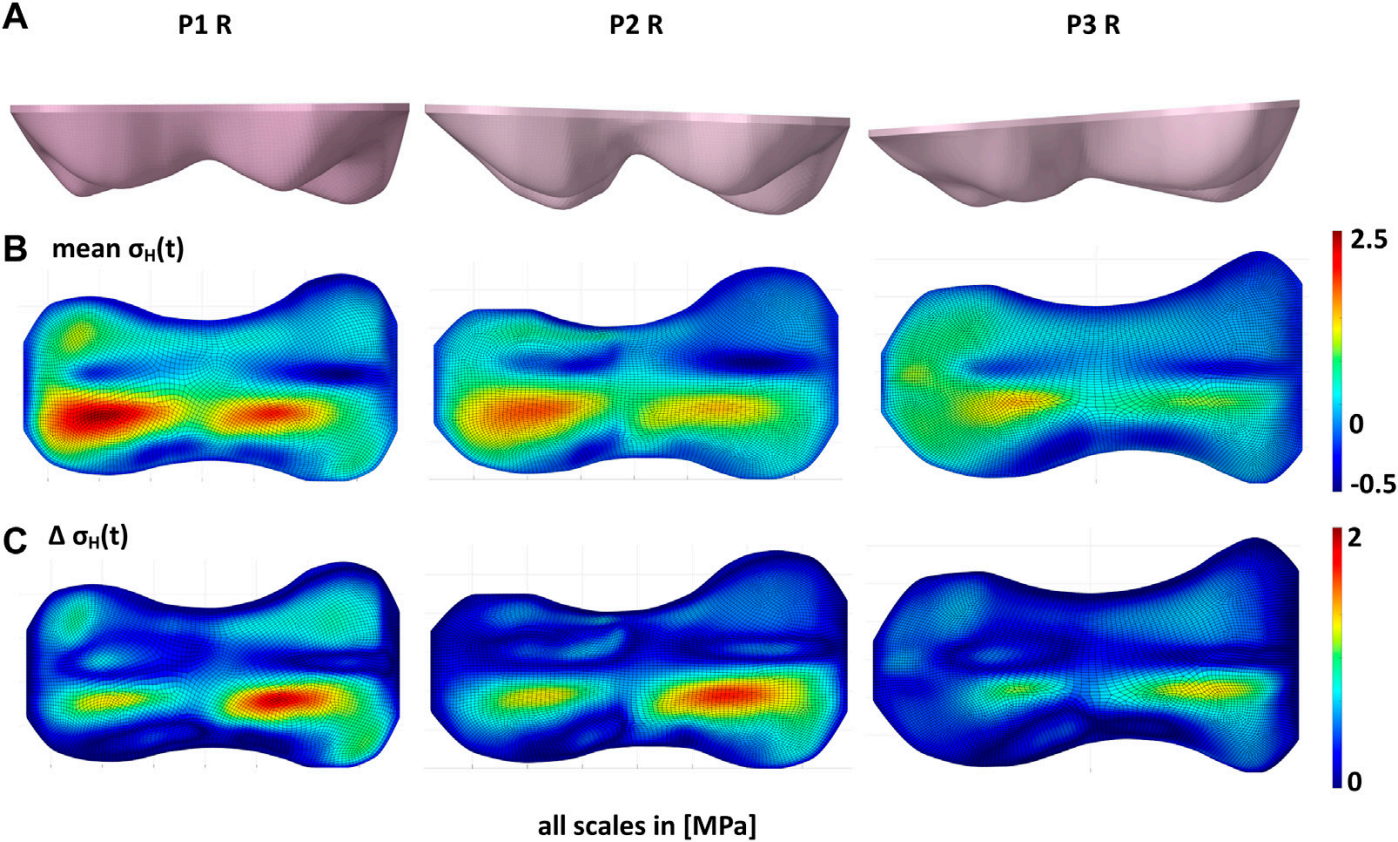
Influence of growth plate geometry on the stress distribution in the growth plate. **(A)** shows the different growth plate geometries of the right knees of patients P1, P2, and P3. With the same loads **(B)** shows that the mean of hydrostatic stresses over the gait cycle ( $\sigma_{H}\left(t\right)$ ) is significantly higher in P1. The same applies to **(C)** the peak-to-peak-amplitudes ( $\sigma_{H}\left(t\right)$ ), although the difference is not so pronounced here.

Simulations show a heterogeneous distribution of stresses over the GP (Figure 4A). In all models that are not affected by pretension of the implant at the end of treatment, the area of the ridges showed the lowest absolute values of $\sigma_{H}$ and $\sigma_{S}$ . Anterior to the medial and lateral ridge, between the ridges and the vertices, the highest pressure stresses occurred, especially during terminal stance. Tension was present especially at the anterior periphery of the GP and posterior to the medial and lateral ridges. The stress distribution in the GP followed the distribution of the external forces. If the medial KCF became very high, this was reflected in increased medial stresses. This was particularly clear in the terminal stance (Figure 4A). Furthermore, the magnitude of stress in the GP was dependent on the GP geometry. When we compared different models with different GP geometries, but the same boundary conditions, the resulting stress distributions qualitatively remained the same between the GPs compared, but the magnitude of the stresses $\sigma_{H}$ and $\sigma_{S}$ changed considerably. This was particularly evident between GPs with different angles relative to the femoral axis (Figure 5).

**FIGURE 6:**
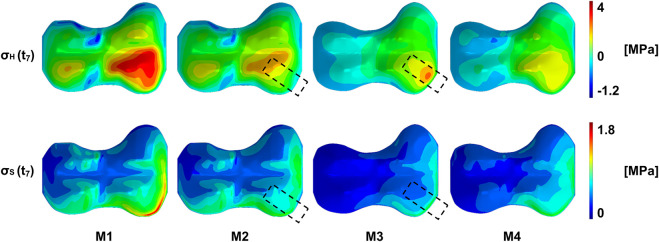
Exemplary representation of the stress distribution of the growth plate of patient P2 R at a point in time in the terminal stance phase. From left to right the same load case is shown at different stages during the treatment: M1/M2–start of treatment without/with implant, respectively, M3/M4–end of treatment without/with prestressed implant, respectively. On top, the pattern of the pressure stresses is shown, with the highest compression stresses in the anterior quadrants. This pattern can be seen, as long as the implant is not under prestresses. Here (M3), high pressure stresses appear in the implant area. After explantation, the original pattern returns with a different distribution of pressure stresses. A similar pattern can be seen on the bottom with the octahedral shear stresses. More detailed figures can be found in the supplementary material.

At the start of the treatment, right after implantation, the overall pattern of stress distribution remained unchanged, but a decrease of the absolute stress values could be observed, on the implant side. This is exemplarily shown in Figure 6 for the terminal stance phase of P2 R. In the case of P1 L and P1 R, the implant reduced the stresses on the entire medial side, with the greatest effect directly at the implant (Supplementary Figures 16, 17). In P2 R and P3 R, the implant was anterior, so the main change was observed directly at the implant, with only small changes in the rest of the medial half (Supplementary Figures 18, 19). At the end of the treatment, when the implant is under pretension, high pressure and shear stresses were observed in the area of the implant. The implant also had an effect on the lateral side, where tension stresses occurred in the whole half of the growth plate (Figure 6, Supplementary Figures 16–23, M3). While the tension stresses in M1 and M2 had a higher magnitude, but where also only occurring locally and depending on the gait phase, tension stresses in M3 covered the whole half of the GP and occurred over the whole gait cycle. After explantation, the initial pattern returned, but with more balanced medial and lateral stresses, and an overall decrease in absolute stress values (Figure 6). The exact pattern depends on the individual geometry and loading conditions.

**FIGURE 7:**
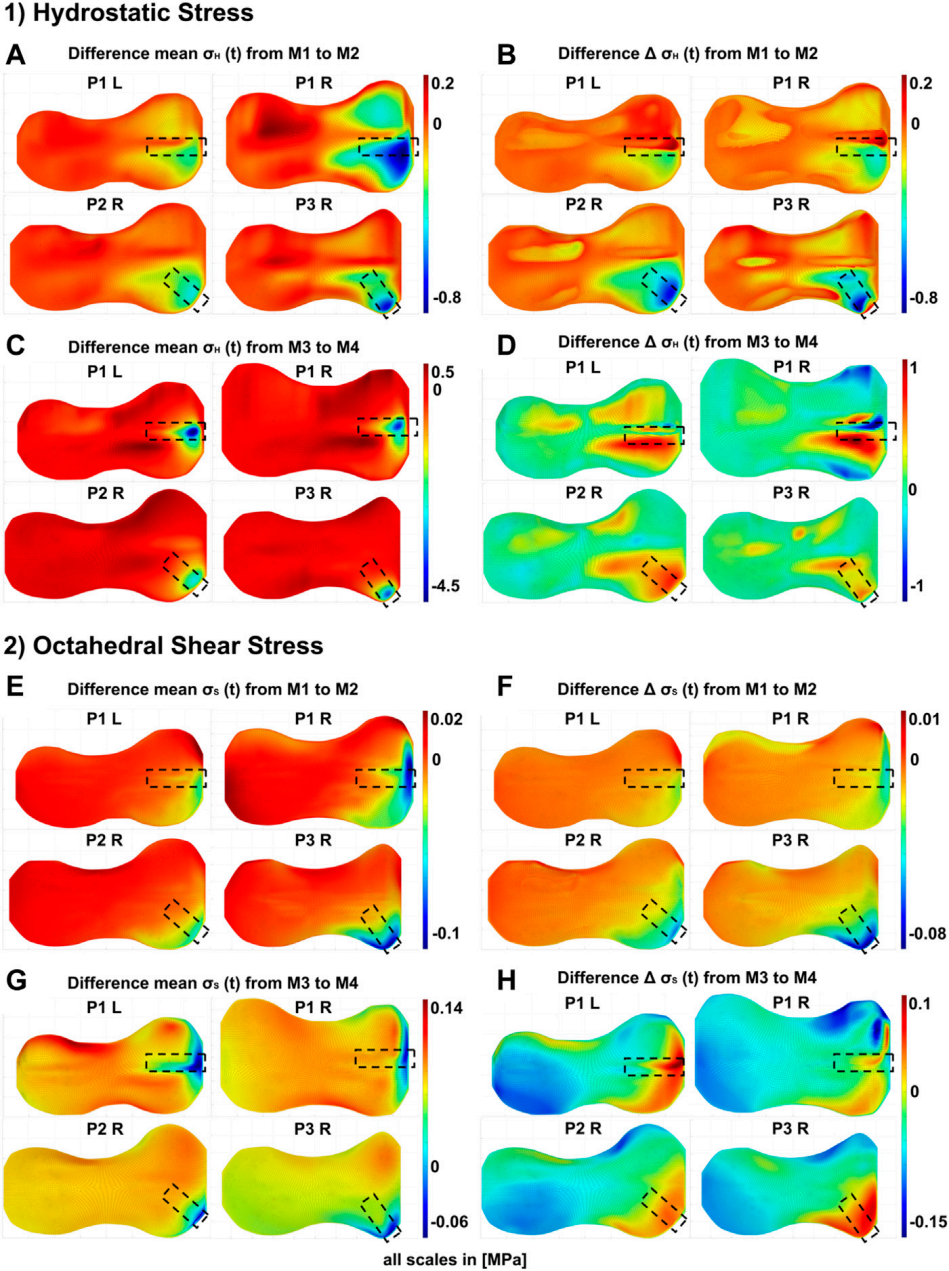
Differences in the GP stress distributions resulting from the presence or absence of the implant at different stages of the treatment: M1/M2–start of treatment without/with implant, respectively, M3/M4–end of treatment without/with prestressed implant, respectively. 1) Differences of hydrostatic stresses in the growth plate; the figure shows the difference of M1 and M2 for each patient (P1 to P3, R = right knee, L = left knee) in **(A)** Peak-to-peak amplitudes $\Delta \sigma_{H}\left(t\right)$ and **(B)** for $mean\;\sigma_{H}\left(t\right)$ . It shows the difference of M3 and M4 for each patient in **(C)** Peak-to-peak amplitudes $\Delta \sigma_{H}\left(t\right)$ and **(D)** for $mean\;\sigma_{H}\left(t\right)$ . 2) Differences of octahedral shear stresses in the growth plate; the figure shows the difference of M1 and M2 for each patient in **(E)** Peak-to-peak amplitudes $\Delta \sigma_{S}\left(t\right)$ and **(F)** for $mean\;\sigma_{S}\left(t\right)$ . It shows the difference of M3 and M4 for each patient in **(G)** Peak-to-peak amplitudes $\Delta \sigma_{S}\left(t\right)$ and **(H)** for $mean\;\sigma_{S}\left(t\right)$ .

Figure 7 shows the changes in the stress distributions that were induced by adding or removal of the implant. At the beginning of the treatment, the implant has the biggest influence in the quadrant where it was placed. Here it reduced the cyclic as well as the static loading. On the contralateral side, the implant resulted in no or small increases in static loading. The cyclic loading was reduced throughout the GP, although significantly less than in the direct area of influence of the implant. Depending on the location of implantation the area of influence is slightly different, although it has the main influence on the anterior medial quadrant and a smaller influence on the posterior medial quadrant (Figures 7A, B, E, F).

At the end of the treatment, as a result of the removal of the implant, pressure and shear stresses where significantly reduced, i.e., the implant induced high pressure and shear stresses locally. Outside the direct area of influence of the implant, either no change or a slight increase with implant removal was observed. The influence on the dynamic stress was more extensive. In P1 L and P1 R, the main changes were seen in the anterior medial quadrant, with some still significant changes in the posterior medial quadrant. In P2 R and P3 R, the implant mainly changed stresses on the anterior medial quadrant. The removal of the implant led to an increase in cyclic change of loading, i.e., the implant decreased the cyclic change of loading during the treatment. Other areas where not changed by implant removal. An exception is P1 R, where the removal of the implant locally decreased the cyclic load changes in small areas. However, the general trend here follows the stress distribution of the other participants.

**FIGURE 8:**
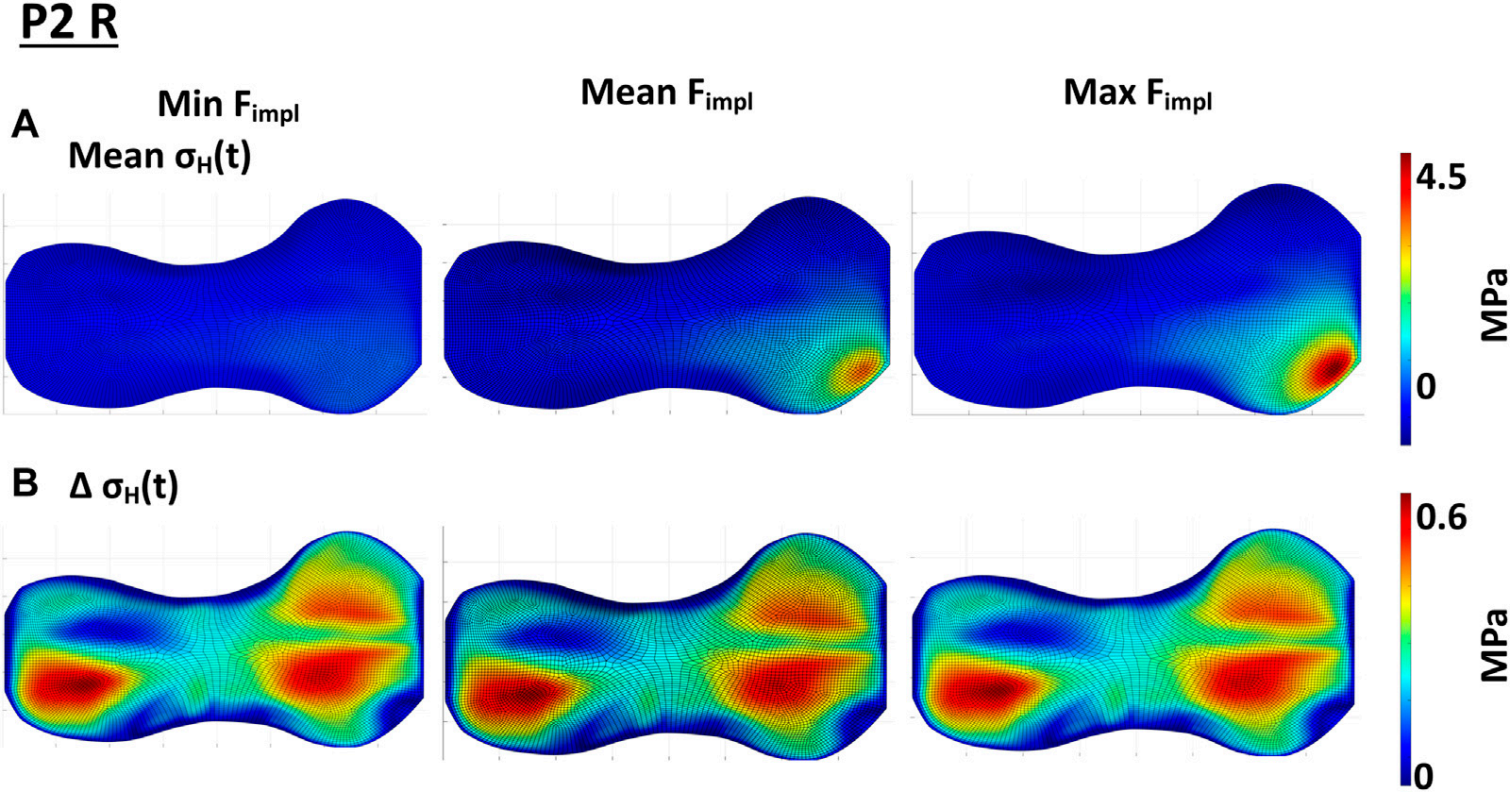
Influence of the implant force on the static compressive stress distribution; **(A)** shows the change in $mean\;\sigma_{H}\left(t\right)$ over the gait cycle when varying the implant force. With the minimal implant force after Schneider et al. (2018) the implant has little to no influence on the stress distribution in the growth plate. The higher the implant force gets, the higher the compressive stress in the growth plate and the bigger the area of influence. **(B)** shows, that the magnitude of the implant force does not have an influence on $\Delta \sigma_{H}\left(t\right)$ over the gait cycle.

The variation of the magnitude of the pretention force exerted by the implant at the end of treatment (cf. Section 2.3.2) influenced on the size of the affected GP area and magnitude of the stresses. Exemplary results for P2 R are shown in Figure 8, the results for all included knees can be found in Supplementary Figures S25–S29. The higher the implant force acting on the GP, the higher are also the local pressure stresses and the bigger the area of influence. While the implant with the minimal implant force only had a small influence on the static loading, the highest implant force also led to the highest static loading of the GP. This effect seemed not to be linear (Figure 8A, Supplementary Figures S24–S27). The magnitude of the implant force showed only negligible influence on the cyclic loading in the GP (Figure 8B, Supplementary Figures S24–S27).

## 4 Discussion

To the best of our knowledge, this is the first numerical finite element study, which used participant-individual load cases combined with a personalized, although not fully individual geometry of the distal femoral GP in order to investigate the heterogenous stress distribution in the GP and the mechanical influence of tension band implants on it in detail.

To evaluate the influence of the tension band implant on the mechanical loading of the juvenile GP, four different models of each of the four different knees were build, representing different stages of the treatment. The model geometries, based on radiographs, were not fully participant-individual, but represented a realistic and characteristic geometry of the distal femoral GP. Boundary conditions and load cases were obtained from instrumented gait analyses of three different participants and subsequent patient-specific musculoskeletal modelling using OpenSim that were available from a previous study (Holder et al., 2020). The goal of this prospective study was to investigate the difference between medial or lateral KCF before and after temporary hemiepiphysiodesis in patients with valgus malalignment compared to a typically developed control group and to determine if a linear relationship exists between the static radiographic mechanical axis angle and the KCFs.

An investigation carried out as part of this study showed that the geometry of the GP has a significant influence on the stress distribution in the GP. Under the same boundary and loading conditions, the stresses in the GP were significantly different for different geometries. This was mainly related to the magnitude of the stresses, rather than the qualitative distribution in the GP, as all GPs showed similar geometrical or morphological features with different degrees of expression.

Due to these recurring geometric features, the stress distributions follow a pattern. The highest pressure stresses were found in both anterior quadrants, close to the medial and lateral ridges, at the steep area. The highest tension stresses were on the other side of the ridges in the posterior quadrants and in the front area of the GP (Figures 4, 5). This indicates that the ridge works as a lever, depending on the joint reaction forces. This assumption is supported by the observation that high pressure stresses in one place in the GP went hand in hand with high tension stresses on the other side of the ridge. This was observed in the diagonal and in the sagittal direction (Figure 4, Supplementary Figures S8–S23). The assumption that the ridges work as a lever was strengthened by a numerical parameter study by Piszczatowski (2012). Eight different geometries and five different arbitrarily chosen load cases were investigated using cylindrical models with the different GP geometries embedded. The RoL was considered as a soft solid body around the cylinder. Although the models were simplified, two load cases in particular, each with two geometries, show similar characteristics to the growth plates we investigated: A wave-shaped growth plate under diagonal loads was evaluated, which was comparable to the simulation of each model in terminal stance. In Piszczatowski (2012) as well as in this study, the stress maxima were observed in the steeply sloping areas of the geometry. On the other side of the ridge, the stress minimum could be identified, suggesting the ridge acts as a lever.

Figure 6 shows the differences in the stress distribution that result from adding or removing the implant, whereas geometry, boundary conditions and external loading remained unchanged. This allowed to isolate the influence of the implant in exemplary cases. Hereby, the peak-to-peak-amplitudes represented the cyclic change of the loading, the mean stresses over the gait cycle showed the static loading. With the insertion of the implant, the cyclic loading and unloading was reduced locally (Figures 7B, 7F), as well as the static compression (Figures 7A, 7E). At the end of the treatment, the prestressed implant reduced the cyclic change of loading and increased the static compressive stresses in the area of the implant (cf. changes between M3 and M4 as shown in Supplementary Figures S8–S15). The effect size regarding the compressive stresses depended on the implant force. The higher the force, the higher the increase in static pressure stresses and the bigger the influence area of the implant (Figure 8). Both, reduced cyclic loading and increased static compressive stresses, were factors that are considered to reduce the growth rate according to Hueter-Volkman (Hüter, 1862a; Huter, 1862b; Volkmann, 1862; Mehlman et al., 1997; Gottliebsen et al., 2016) and Frost (Frost, 1979). These findings confirm our initial hypothesis stated above (cf. Section 1). Surprisingly, the implant initially reduced the static loads on the GP since part of the loading was transferred from epiphysis to metaphysis via the plate instead of the GP. Since it also reduced the cyclic loading at the beginning, a growth inhibiting and a growth stimulating factor were observed for this stage of treatment.

The implant did not only have an impact on the stress distribution on the joint side where it was placed. Right before explantation of the prestressed implant an increase in tension stresses on the contralateral side of the GP was observed. Since tension stresses are believed to increase the growth rate, this would mean, that axis correction by the implant is not only achieved by suppressing growth on one side, but also by promoting growth on the other side of the GP.

Few studies have been conducted using FE modelling to understand the influence of the mechanical loading of the respective GP and the surrounding structures on the stress distribution in the GP. Hereby, mainly growth models were investigated, all of them used different versions of the OI to connect the mechanical loading with the resulting growth. Carter und Wong (1988) studied the role of mechanical loading in development of diarthrodial joints in human fetuses, with the aim to develop a hypothesis on how to describe the behavior of the GP tissue sufficiently. Therefore, simplified geometries and load cases were used in the FE-model, which consisted of the joint bones and cartilage of infantile subjects. In contrast to the current study, perichondrial structures were not included. A constructed gait cycle was simulated as discrete load steps and the contact forces were applied on nodes in the contact area of the joint, as was also done in this study. Since the fluid flow between the cartilage and the growth plate is slow, the assumption of a linear elastic, nearly incompressible model was considered sufficient to investigate the influence of mechanical loads on the tissue. The same material model was applied in this study. The model predicted the ossification center and the development of the growth plate in infantile joints.

Carriero et al. (2011) used a similar approach and investigated the effect of altered gait due to cerebral palsy on the proximal femoral morphology in later developmental stages. Here, transition zones with gradually increasing Young´s Modulus from the GP to the bone were added to improve the simulation stability, as we did in our model. They also make sense when compared to the biological structure, as the GP becomes harder gradually across its layers.

Yadav et al. (2017) studied the influence of muscle groups on the proximal femoral growth in able bodied children between the ages of 6 and 11 years old. Using previously described methods the study used nine load cases representing the stance phase of the gait cycle, as was also done in this study. The direction of growth was considered in the direction of the maximum principal stress for each element and the growth was applied by thermal expansion of the outer growth plate layer. Although a model of the whole femur was studied, the distal femoral GP was not included in the model. Only the influence of muscles in healthy children was investigated.

Close to the aforementioned studies Sadeghian et al. (2021) investigated the endochondral ossification in long bones using a phalanx joint. A distinction between calcified and non-calcified cartilage was made. This model could predict the ossification of postnatal cartilage in the phalanx joint.

None of the discussed studies investigated the distal femoral GP in adolescents. Since the geometry of the GP is highly irregular our results are hard to compare to these studies. To investigate the connection between mechanical loading and growth all aforementioned studies used the OI, but all in a different way. The OI always consists of the octahedral shear stress as the growth increasing factor and the hydrostatic stress as the growth inhibiting factor. Carter und Wong (1988) used the sum of OI over all load cases and first proposed an experimental constant weighting of the hydrostatic stress. Sadeghian et al. (2021) used the same approach but calculated the OI for each element in each load step and used the mean OI over all load steps for each element. Carriero et al. (2011) and Yadav et al. (2017) each used the minimum hydrostatic stress and maximum octahedral shear stress over all load cases and used a weighting factor for both stresses, so that the mechanical contribution to growth is half as the biological contribution. Piszczatowski (2011) used two different approaches of the OI and the main principal stresses to evaluate the results. Since formulation and use of the OI for predicting bone growth is inconsistent and not validated, we decided to restrict this study to the evaluation of the distributions of compressive and deviatoric stresses. Future work of our group will aim at validation of the OI for adolescents. Furthermore, none of the studies differentiated between static and cyclic compression.

In contrast to the aforementioned studies, part of our model was the RoL. It is part of the perichondrium and encloses the GP in the circumferential direction. Biologically, the circumferential growth of the bone originates from these structures, therefore they also contain stem cells. Mechanically, it prevents the growth plate from being pushed out of the bone and reduces high stresses at the edges. A short numerical sensitivity study was conducted to show the influence of the RoL and the transitions zones. This can be found in the supplementary material, Section 2 and Supplementary Figures S6, S7.

Castro-Abril et al. (2015) investigated the influence of the geometry and force application on the stress distribution in the proximal femoral growth plate and how this influenced a slipped capital femoral epiphysis. To do so, a numerical parameter study was conducted, varying the physeal-diaphysis angle, body mass, RoL, physical activity and GP thickness. The RoL was modelled according to Piszczatowski (2012). Castro-Abril et al. (2015) was able to show whether or not the RoL is considered has a greater influence on the stress distribution and thereby also the growth, than the position and shape of the growth plate or the force application. Due to the complex geometry of the distal femoral growth plate, this cannot necessarily be transferred to our model, but it was shown that the RoL should not be neglected in a mechanical investigation of the structure.

Schneider et al. (2018) conducted the first study investigating the implant and its screws, but did not investigate the effect of the implant on the stress distribution in the GP. They used the screw bending observed on radiographed images at the end of the guided growth treatment to determine the resulting forces on the implant. Although the validation model used was geometrically simplified, the bending and the stress curve in the screws could be represented well. In comparison to this study, the implant screws showed qualitatively the same bending as in our model and thus are likely to be able to represent the loading of the GP induced by the implant.

### 4.1 Limitations

As already stated above, the exemplarily presented models were not fully participant-individual, meaning that the calculated stress distributions did not allow for the prediction of future growth in the individual case. Geometrical parameters in the frontal plane were measured in the radiograph images, parameters in the transversal and sagittal plane were added from age matched MRI-data (Lustig und Vasanawala, 2022). This led to not fully participant-specific models of the distal femoral GP, which however incorporated typical morphological features and had a realistic geometrical complexity. Due to this geometry, the computed stress patterns were heterogeneous and contained not only pressure, but also tension stresses, although the applied forces were solely compression forces. The material properties of the models were not participant-specific. In contrast, linear elastic properties with parameters based on literature were used. Also, the implant forces applied to the implant in Model M3 were not participant-specific. Instead the mean, minimum and maximum value of the calculated screw forces by Schneider et al. (2018) were used. The positioning of the implant inside the model was based on radiograph images in the frontal plane and thus the parameters in the transversal plane had to be estimated. Within the RoL lies Ranvier’s Groove, which was not considered in our model.

The developed model is a participant-specific but not a participant-individual model. It can be used to investigate the typical effect of tension-band plates on the stress distribution in the growth plate, but it does not allow predictions about the course of treatment or the occurrence of a rebound effect after treatment in the individual case. Future work will include fully patient-specific modelling.

## 5 Conclusion

The current study demonstrates the capability of finite element analyses that model the interplay of the complex GP morphology and realistic loading conditions from experimental gait analysis to show stress distribution in the GP and the implant’s influence, for a given geometry and given material parameters. With untouched loading and geometry between two models, the insertion/exertion of the implant changes the stress distribution in the GP drastically. In order to allow for clinical predictions, these models have to be fully participant-individual with regard to GP morphology and load cases. This together with patient examinations during and after the treatment can show, if these models will be able to determine the implant removal time point, predict further growth, and estimate the risk of the occurrence of rebound.

## Data Availability

The data analyzed in this study is subject to the following licenses/restrictions: Clinical data of patients under the age of 18 years, generated by clinical partner (see authors list). Requests to access these datasets should be directed to FS, felix.stief@kgu.de.

## Abbreviations

FE, Finite Element; GP, Growth plate; KCF, Knee contact force; OI, Osteogenic Index; RoL, Ring of Lacroix.
