# Supplementary figures and images for: Influence of tension-band plates on the mechanical loading of the femoral growth plate during guided growth due to coronal plane deformities
Source: Front Bioeng Biotechnol. 2023 Jun 21;11:1165963. doi: 10.3389/fbioe.2023.1165963 (PMC10321528; doi:10.3389/fbioe.2023.1165963)

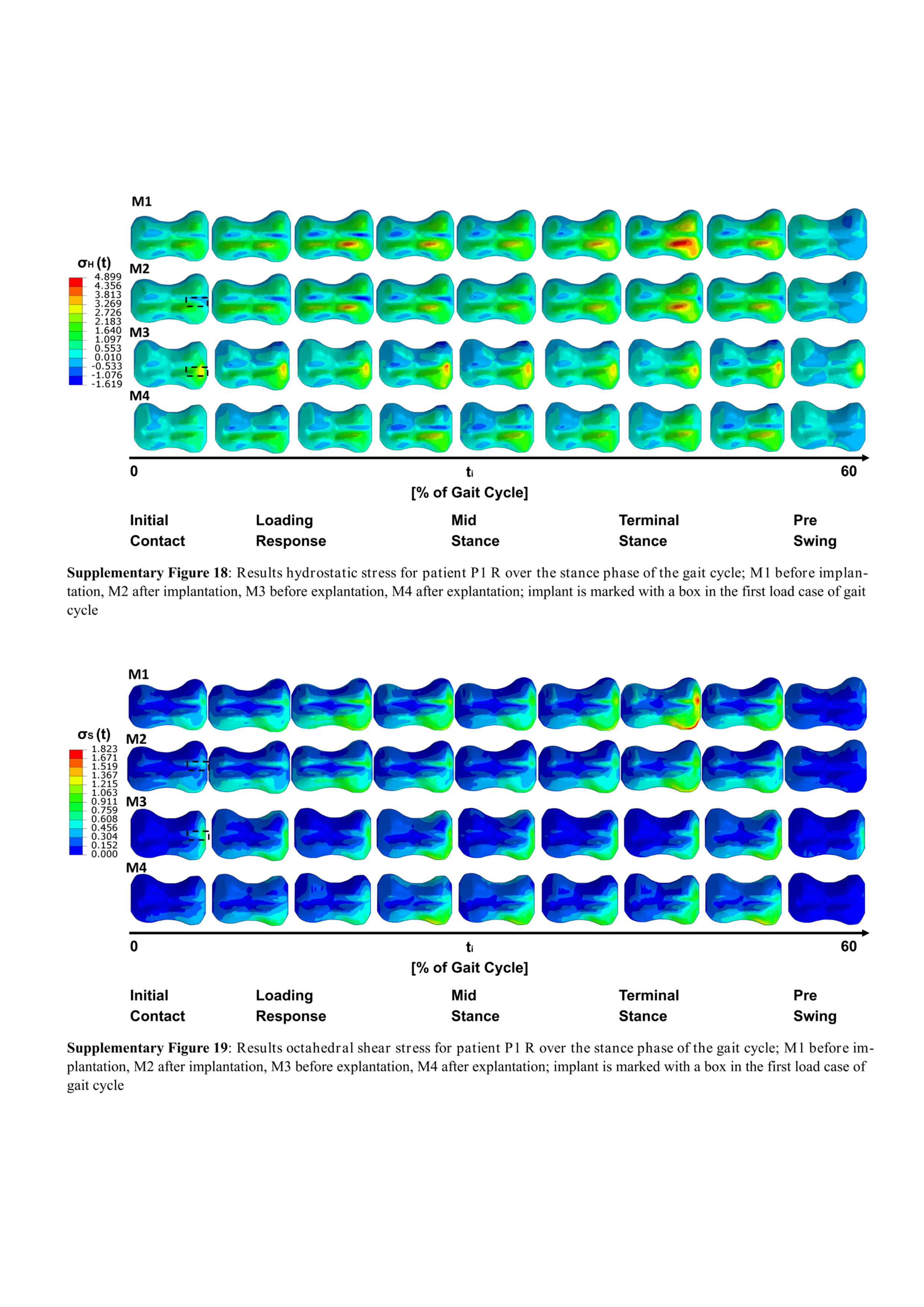

Supplement: Supplementary file 1 [file Image6.TIF]

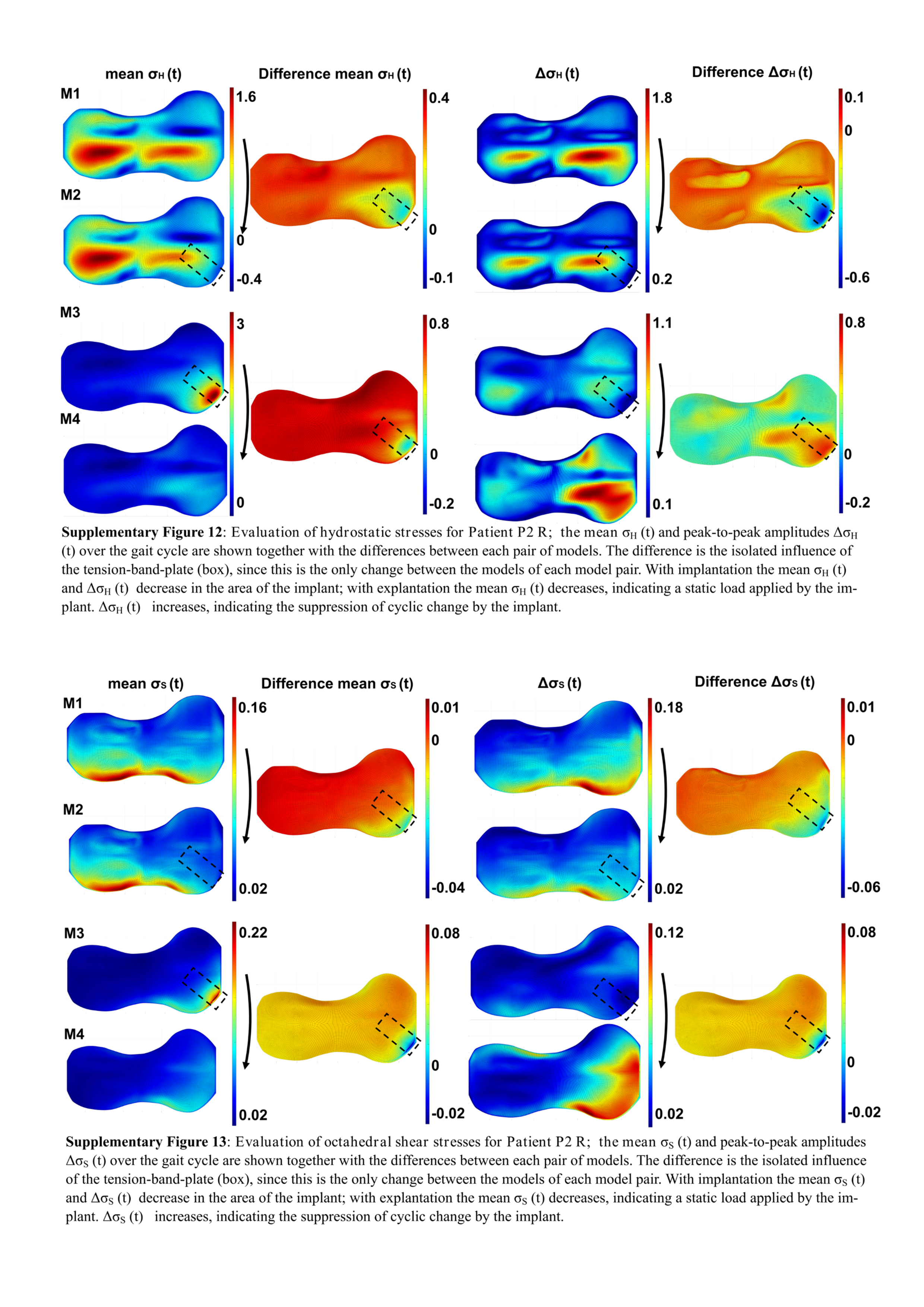

Supplement: Supplementary file 2 [file Image3.TIF]

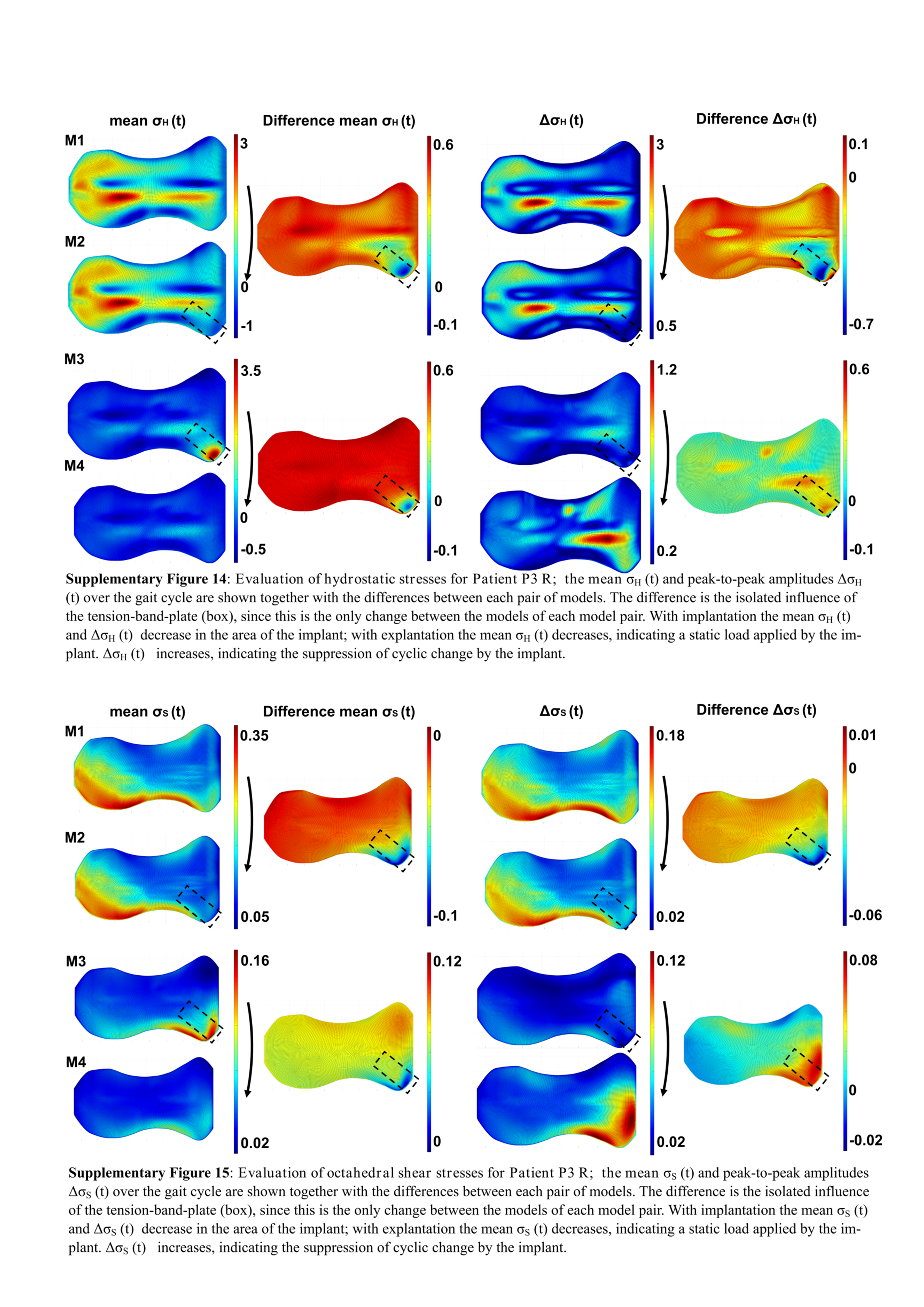

Supplement: Supplementary file 3 [file Image4.TIF]

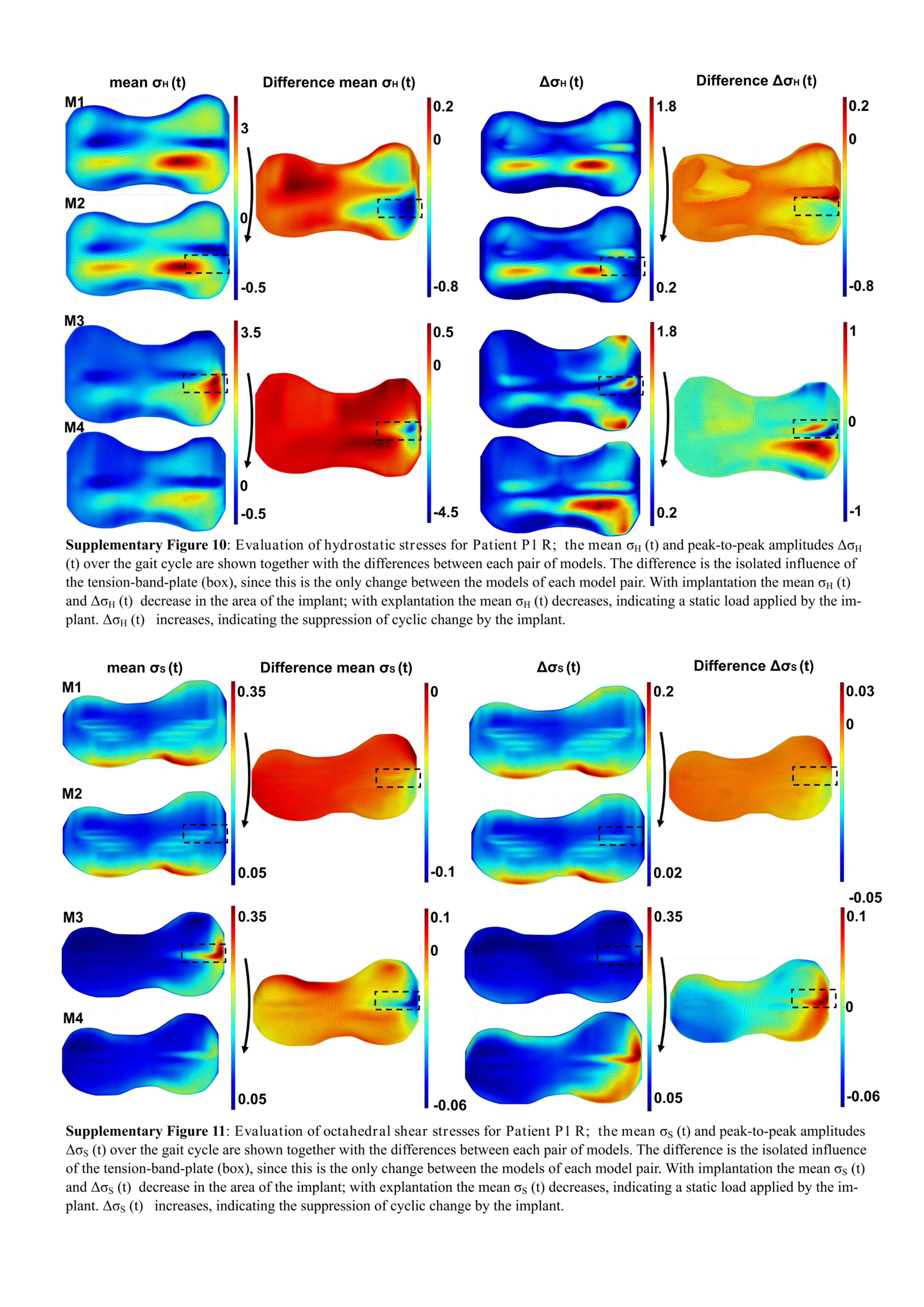

Supplement: Supplementary file 4 [file Image2.TIF]

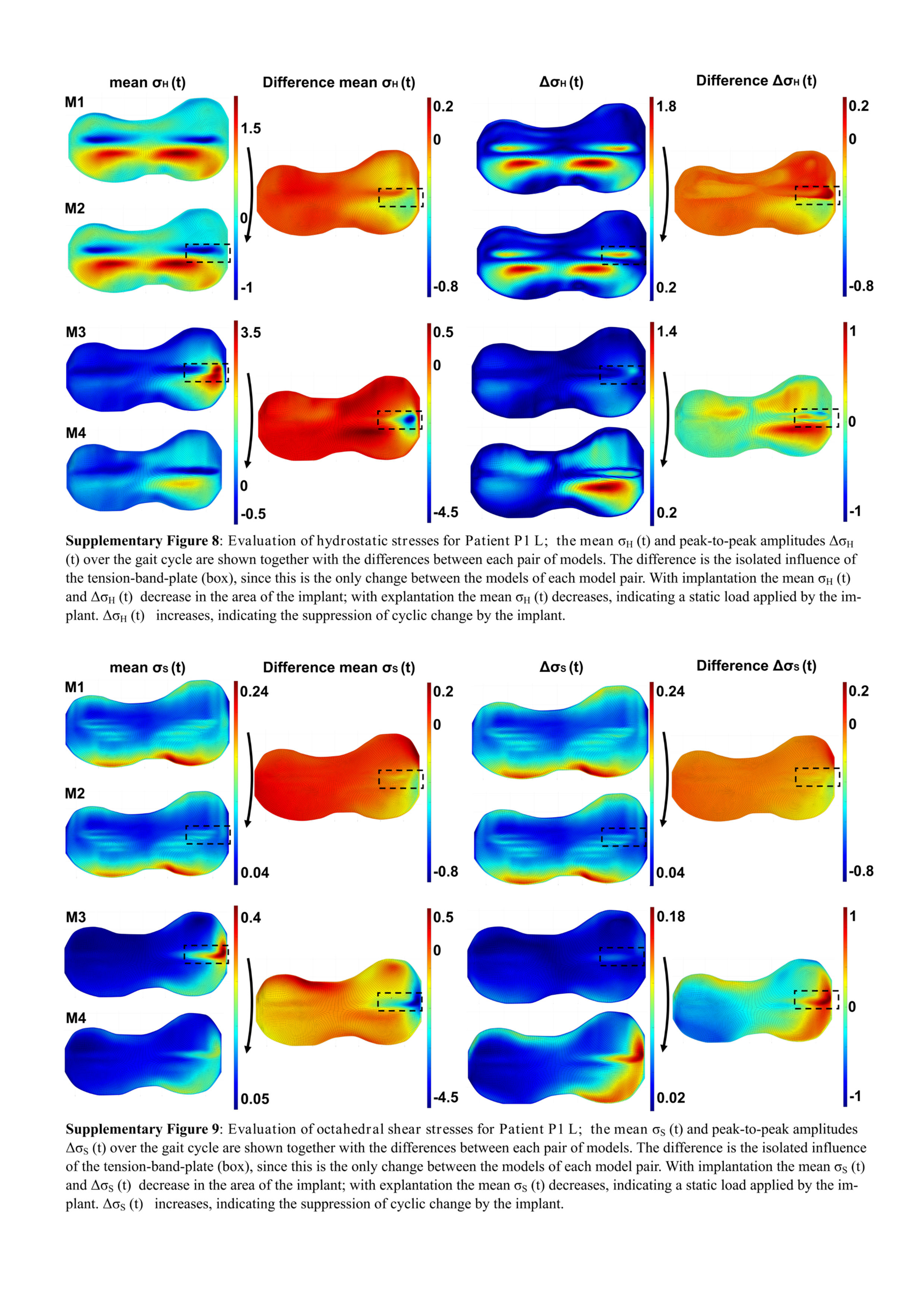

Supplement: Supplementary file 5 [file Image1.TIF]

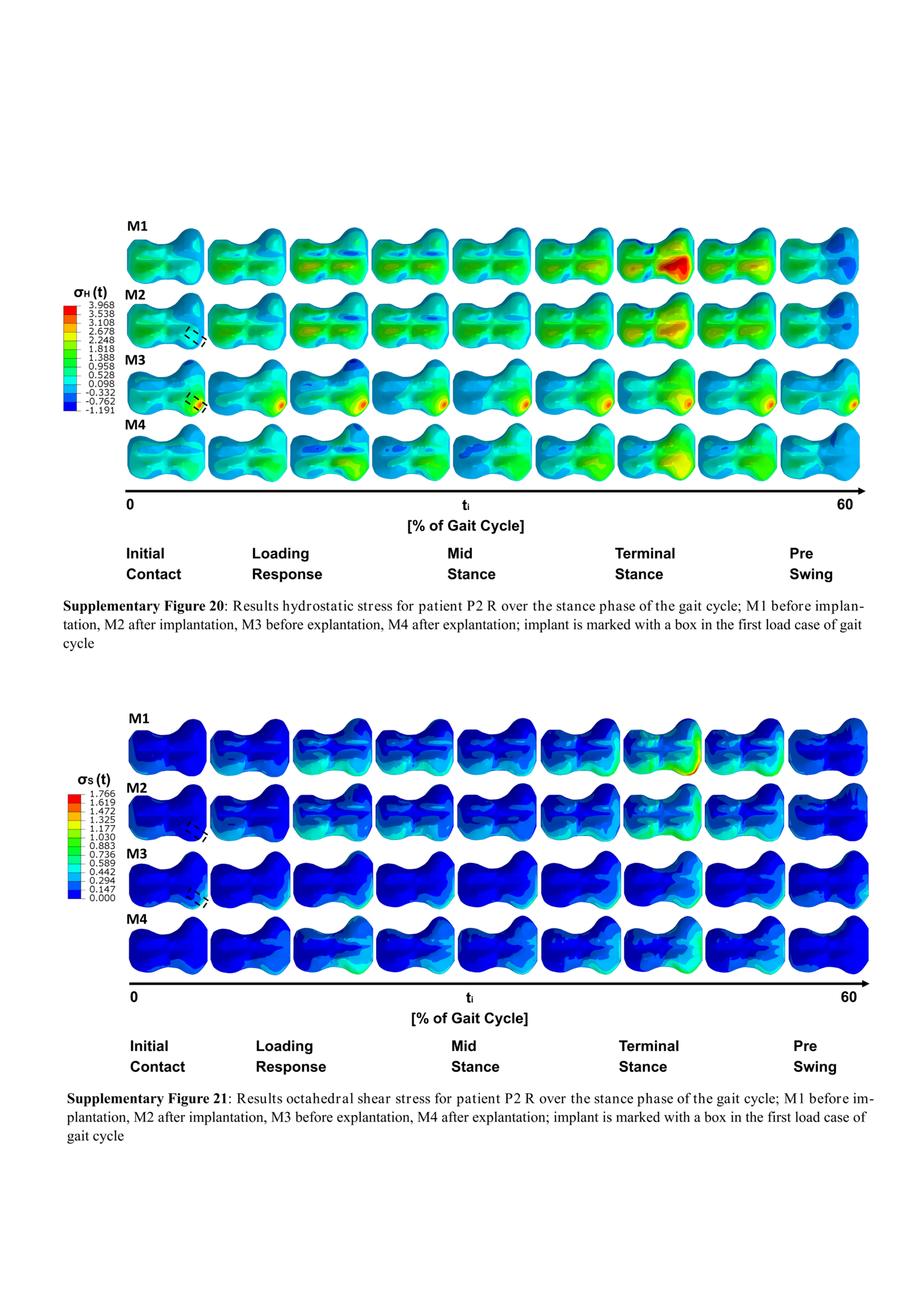

Supplement: Supplementary file 6 [file Image7.TIF]

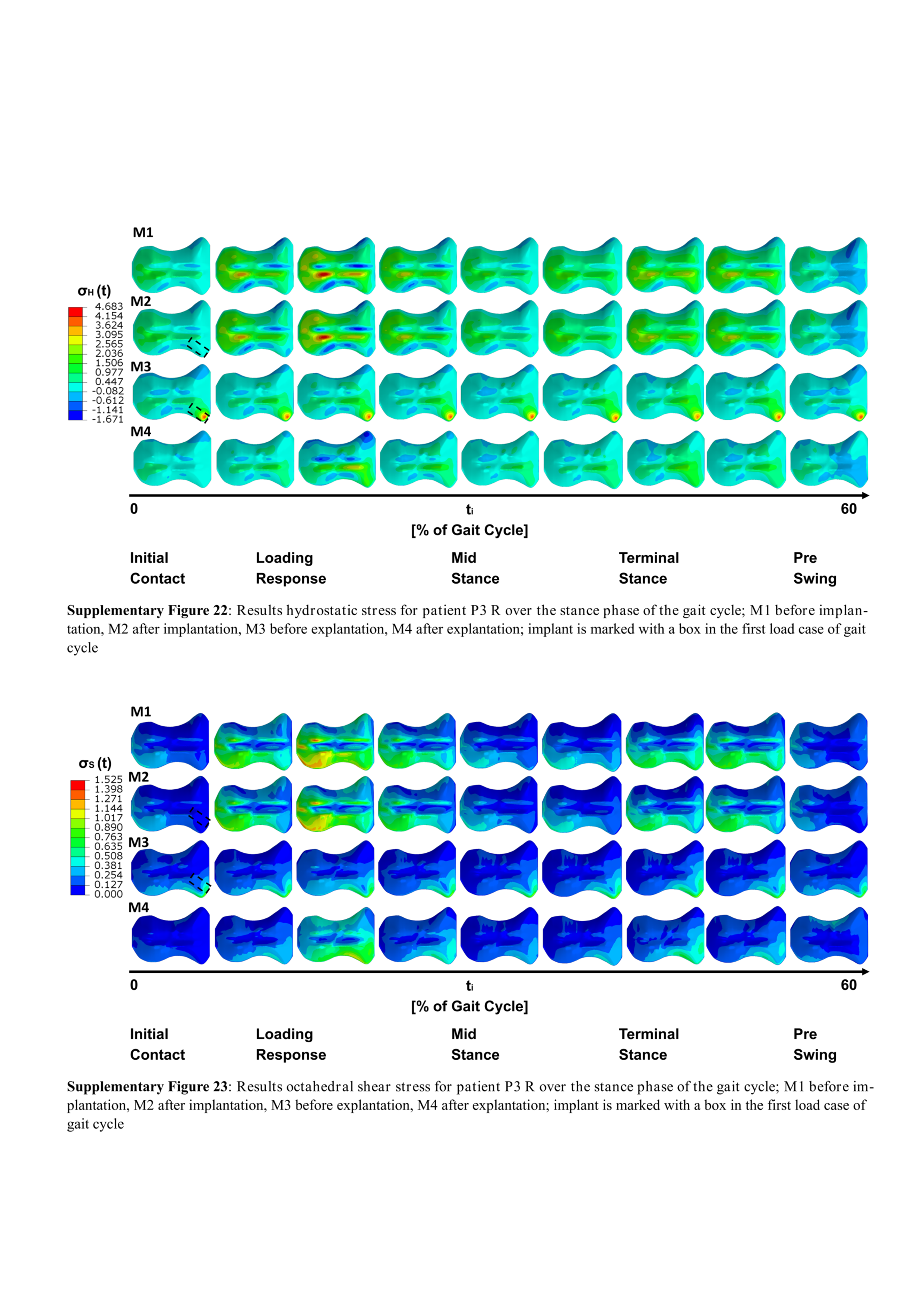

Supplement: Supplementary file 8 [file Image8.TIF]

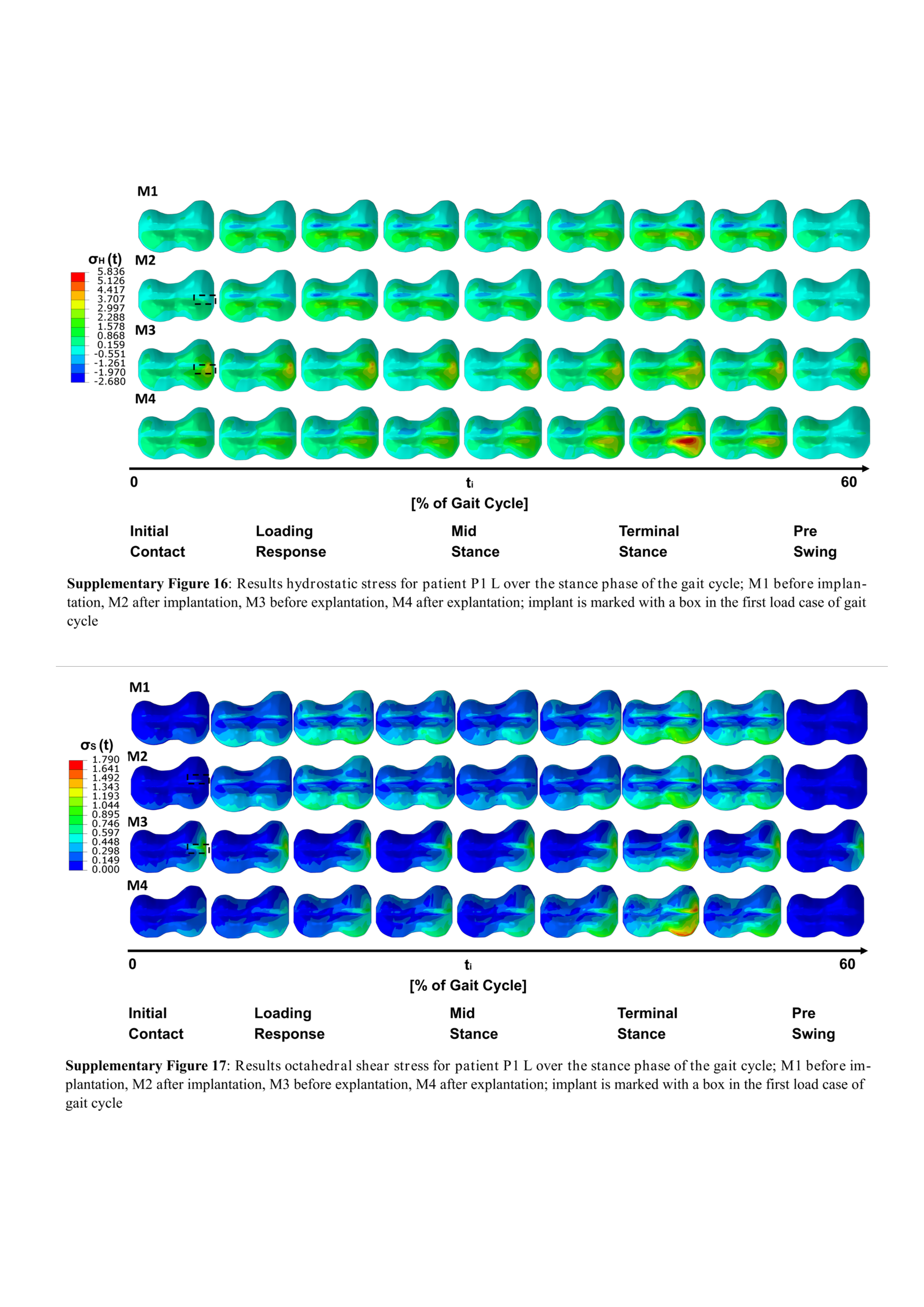

Supplement: Supplementary file 9 [file Image5.TIF]
