# Supplementary material for: Influence of tension-band plates on the mechanical loading of the femoral growth plate during guided growth due to coronal plane deformities
Source: Front Bioeng Biotechnol. 2023 Jun 21;11:1165963. doi: 10.3389/fbioe.2023.1165963 (PMC10321528; doi:10.3389/fbioe.2023.1165963)
Supplement: Supplementary file 7 [file DataSheet1.PDF]

Supplementary Material

1 Convergence Study to determine the appropriate mesh size for the growth plate

A convergence study was carried out to investigate a suitable mesh size and element type. This was done with a section of the original model, consisting of the trabecular bone, growth plate (GP) and transition zones (TZ) (Supplementary Supplementary Figure 1).

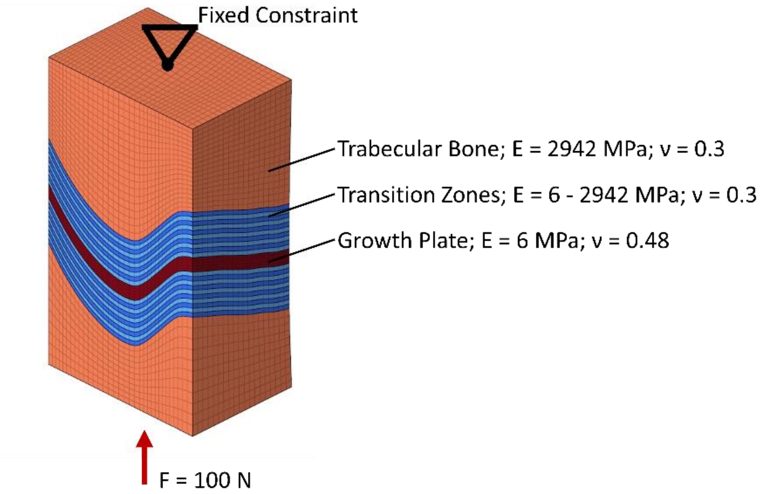

Supplementary Figure 1: Model for the convergence study, consisting of trabecular bone, transition zones and the growth plate; model is fixed in all degrees of freedom at the nodes of the proximal plane, a concentrated force of 100 N is applied, equally distributed on the nodes of the distal plane.

For the study different mesh sizes from 0.1 to 2.0 mm and mesh types were investigated. The used element type was in all cases 8-node-brick elements (C3D8, Abaqus 6.14, Dassault Systèmes Simulia Corp., Vélizy-Villacoublay, France). (Supplementary Table 1)

Table 1: Convergence study; in the same model (Supplementary Figure **1**) the mesh size and mesh type were varied using 8-node-brick elements (C3D8) in all cases and the minimal principal stress and the tresca stress was evaluated.

Mesh Size	Mesh Type	Evaluation Parameters
0.1	First order elements	Minimal principal stress (SP 3)
0.2	First order incompatible mode elements	Tresca Stress

0.3	Second order elements	
0.5	Second order incompatible mode elements	
0.75		
1.0		
1.5		
2.0		

For evaluation of the results of the convergence study the minimal principal stress (SP 3) and tresca stress were evaluated along three different node paths in the GP (Supplementary Figure 2) and also according to the maximum values at any place inside the model. The minimal principal stress was chosen, since the absolute values were the highest because the GP experiences mainly compression stresses.

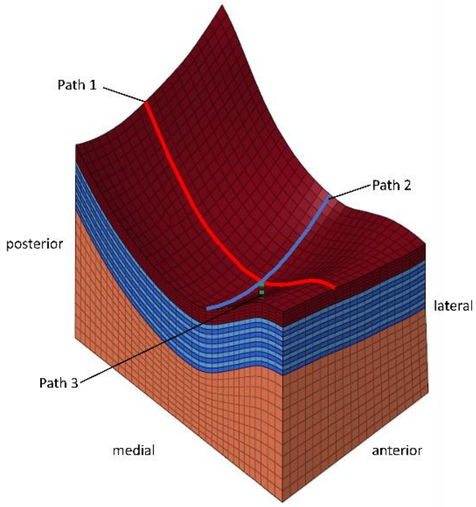

Supplementary Figure 2: Evaluation paths on the growth plate, with anatomical directions. Path one goes along the length of the growth plate, path two the width and path three along the thickness were path one and two are intersecting.

The stress distribution in the growth plate of the model was qualitatively the same in all models. Next evaluated were the different mesh types. Therefore, the different mesh types in the same mesh size were compared. This showed only small deviations, so the mesh type with the lowest calculation time was chosen, which the first order mesh was. The following evaluations were conducted only with the results of the first order models.

Supplementary Figure 3 shows that there is no significant difference in the minimal SP 3 and the maximum tresca stress values anywhere in the model.

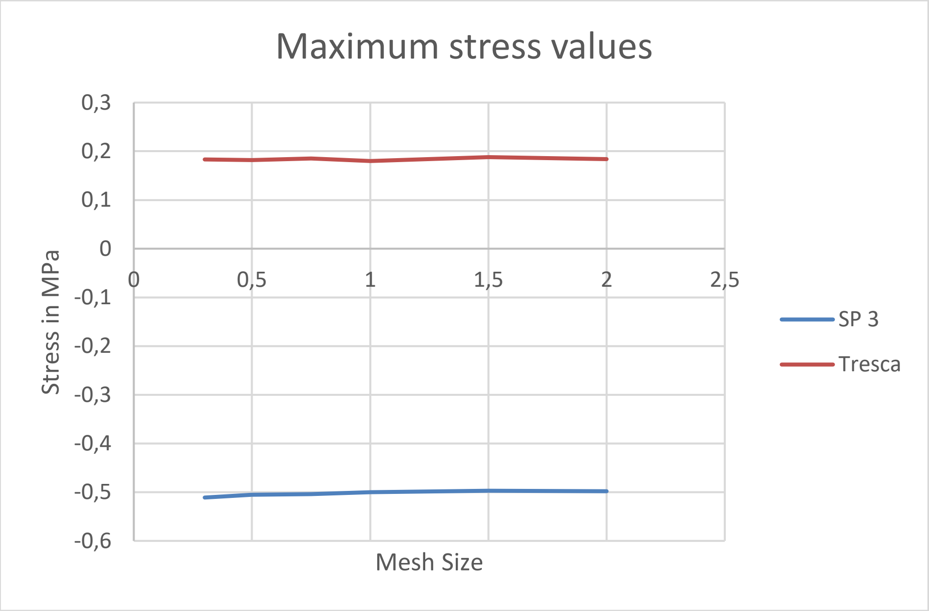

Supplementary Figure 3: The minimal values of the minimal principal stresses (SP 3) and maximal values of tresca stresses are compared over the mesh size.

The main differences in the stress values over the paths, which then lead to the selection of the further used element size, showed the evaluation of SP 3 over the thickness of the GP (Supplementary Figure 4) and the tresca stress over the width of the GP (Supplementary Figure 5). For clarity of the presentation of the results, Supplementary Figure 4 and Supplementary Figure 5 are the only figures shown, although the evaluation was carried out for all different mesh sizes and paths.

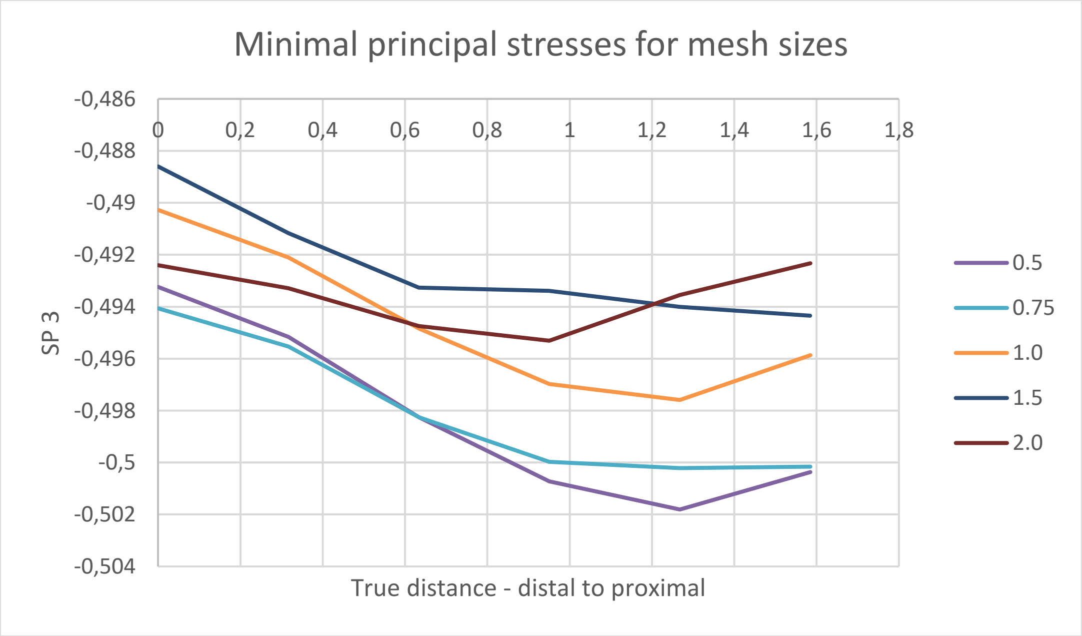

Supplementary Figure 4: Minimal principal stresses (SP 3) of the different mesh sizes over the thickness (path three) of the growth plate (from distal to proximal)

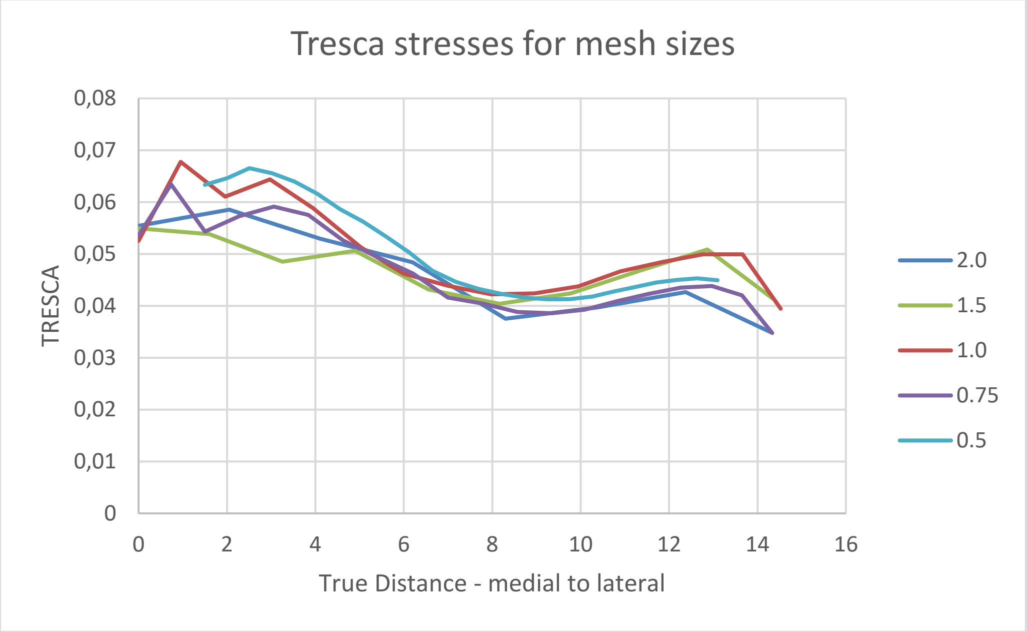

Supplementary Figure 5: Tresca stresses of the different mesh sizes over the width (path two) of the growth plate (from medial to lateral)

The result of the convergence study was, that a first order mesh with the size of 0.75 mm is the best compromise between running time and simulation stability and accuracy of results, therefore the meshing of the model of the whole growth plate was done with this element type and size.

2 Sensitivity study on model components

To show the influence of the Ring of Lacroix and the transition zones on the stress distribution of the growth plate, a short sensitivity study was conducted. Here the same model (Figure 3, M1) was used, but with different components active in each simulation (Supplementary Table 2).

Supplementary Table 2: Model configuration for sensitivity study of components

	Ring of Lacroix	Transition zones
Model 1	Yes	Yes
Model 2	Yes	No
Model 3	No	Yes

Model 4	No	No

The results of the different simulations showed, that the Ring of Lacroix and the transition zones have an influence on the stress distribution, as shown in Supplementary Figure 6. Especially at the edge of the growth plate the influence of the components is visible. Here the Ring of Lacroix reduces the peak stresses, for when the Ring is considered, the stress in the edge area is more evenly distributed. (Supplementary Figure 6, Model 1 and 2) The transition zones have a smaller influence on the stress distribution than the ring in the growth plate, but assure a stable simulation (Supplementary Figure 6, Model 3 and 4).

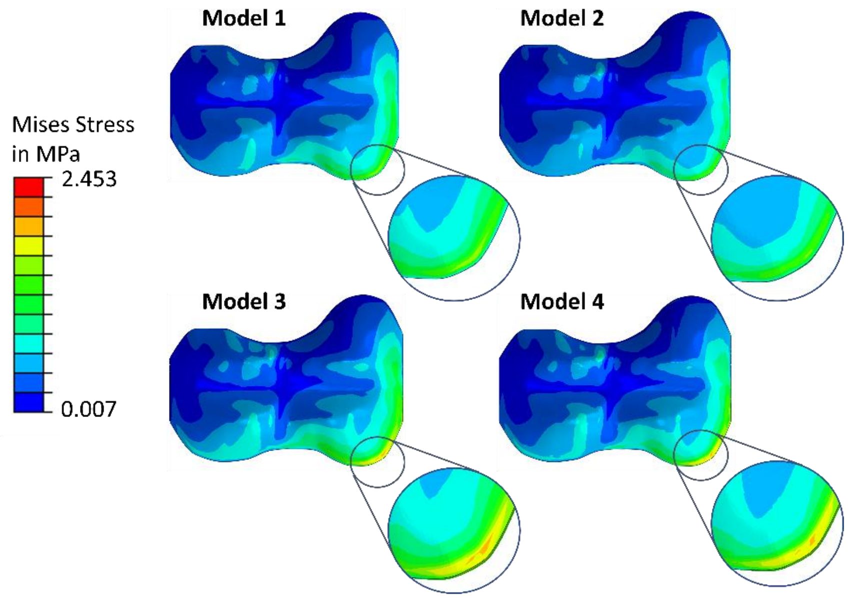

Supplementary Figure 6: Results of study; Mises stresses for the simulation with the Ring of Lacroix and the transition zones (Model 1); with Ring of Lacroix, but without the transition zones (Model 2), without the Ring of Lacroix, but with transition zones (Model 3) and without both components (Model 4)

Since the material properties are linear elastic, the stress is directly linked to the strain of the growth plate. Here the models without the Ring of Lacroix show a higher deformation than the ones without (Supplementary Figure 7, Model 1 and 2). The transition zones again have a smaller impact on the deformation (Supplementary Figure 7, Model 3 and 4).

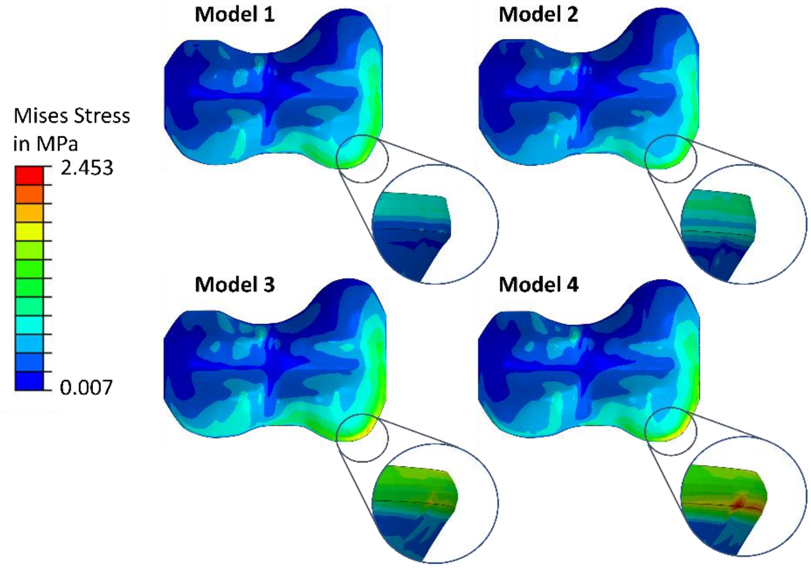

Supplementary Figure 7: Strain of Growth plate due to component change of model; magnified to 2 times the strain; Mises stresses for the simulation with the Ring of Lacroix and the transition zones (Model 1); with Ring of Lacroix, but without the transition zones (Model 2), without the Ring of Lacroix, but with transition zones (Model 3) and without both components (Model 4)

3 Evaluation of simulation results

For evaluations of simulation results for each patient, see supplementary figures 8 – 15.

4 Simulation results

For all simulation results for each patient, see supplementary figures 16 – 23.

5 Influence of the implant force on the screws

The figures show the results of the small parameter study, which was conducted to show the influence of the implant force acting on the screws in Model M3. The approach is described in the manuscript Chapter 2.3.

P1 L

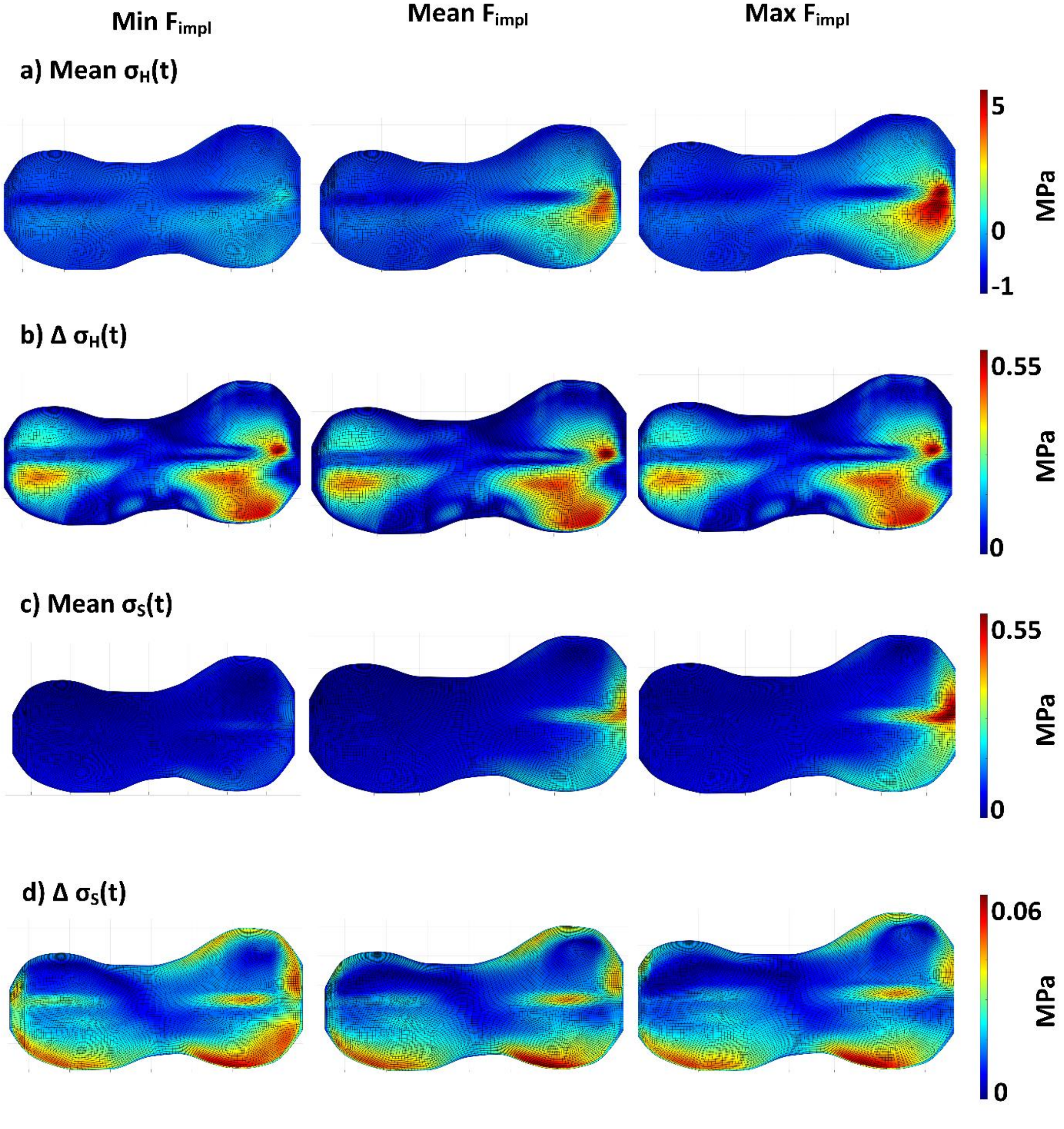

Supplementary Figure 24: Results of the minimum, mean and maximum implant force as boundary conditions on model M3 of patient **P1 L**. Sorted in Columns, the first column shows the minimum implant force (Min F_{impl}), the second shows the mean (Mean F_{impl}), and the third shows the maximum (Max F_{impl}). a) shows the results for the mean hydrostatic stresses over the gait cycle (Mean $\sigma_H(t)$). While at the minimum implant force there is little to no static pressure in the growth plate, with higher implant forces, the magnitude of the static pressure gets higher. The same applies to c) the mean octahedral shear stresses (Mean $\sigma_H(t)$). The peak-to-peak-amplitude over the gait cycle is not significantly influenced by the implant forces, as shown in b) for the hydrostatic stresses ($\Delta\ \sigma_H(t)$) and d) for the octahedral shear stresses ($\Delta\ \sigma_H(t)$).

P1 R

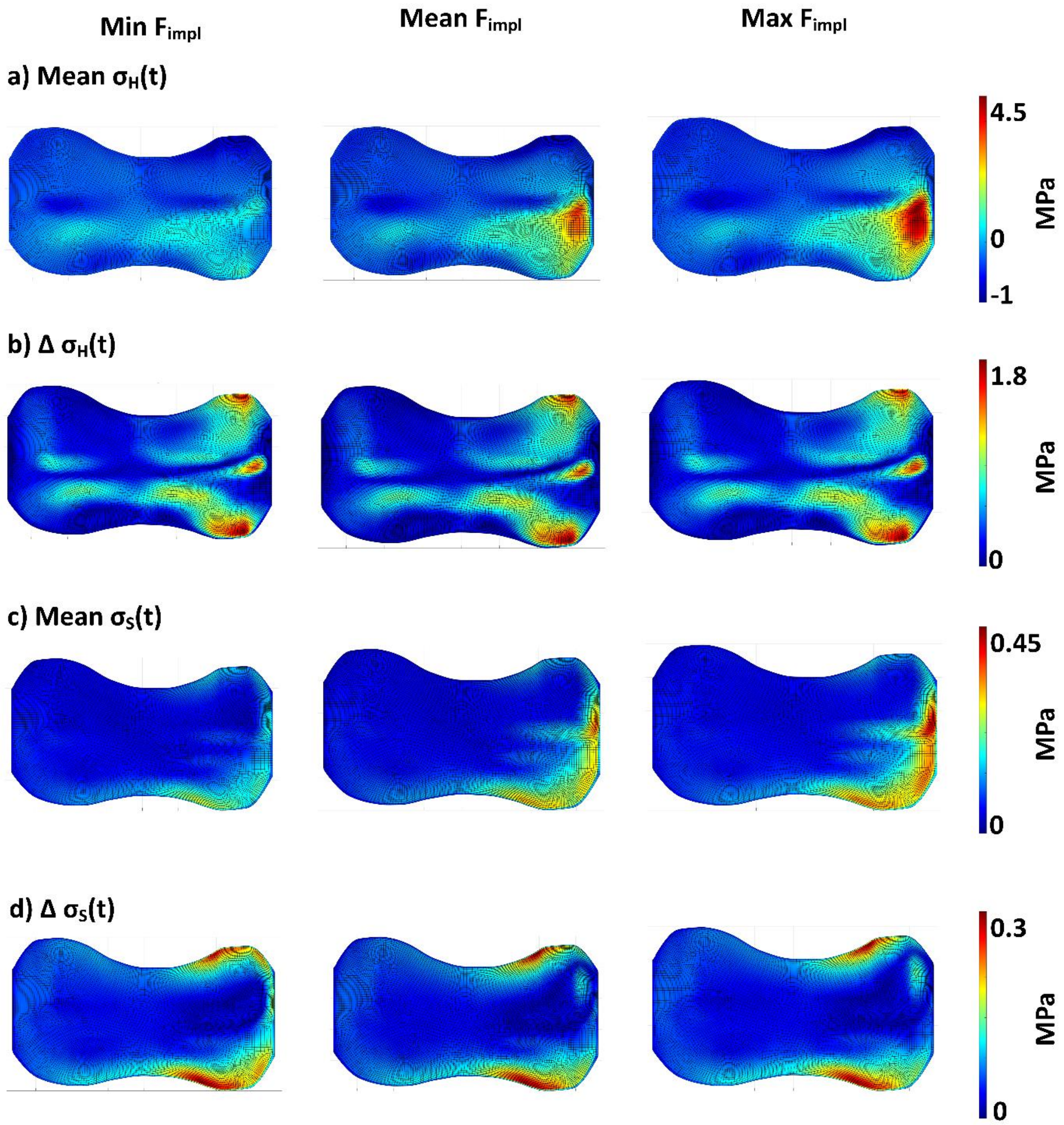

Supplementary Figure 25: Results of the minimum, mean and maximum implant force as boundary conditions on model M3 of patient **P1 R**. Sorted in Columns, the first column shows the minimum implant force (Min F_{impl}), the second shows the mean (Mean F_{impl}), and the third shows the maximum (Max F_{impl}). a) shows the results for the mean hydrostatic stresses over the gait cycle (Mean $\sigma_H(t)$). While at the minimum implant force there is little to no static pressure in the growth plate, with higher implant forces, the magnitude of the static pressure gets higher. The same applies to c) the mean octahedral shear stresses (Mean $\sigma_H(t)$). The peak-to-peak-amplitude over the gait cycle is not significantly influenced by the implant forces, as shown in b) for the hydrostatic stresses ($\Delta\ \sigma_H(t)$) and d) for the octahedral shear stresses ($\Delta\ \sigma_H(t)$).

P2 R

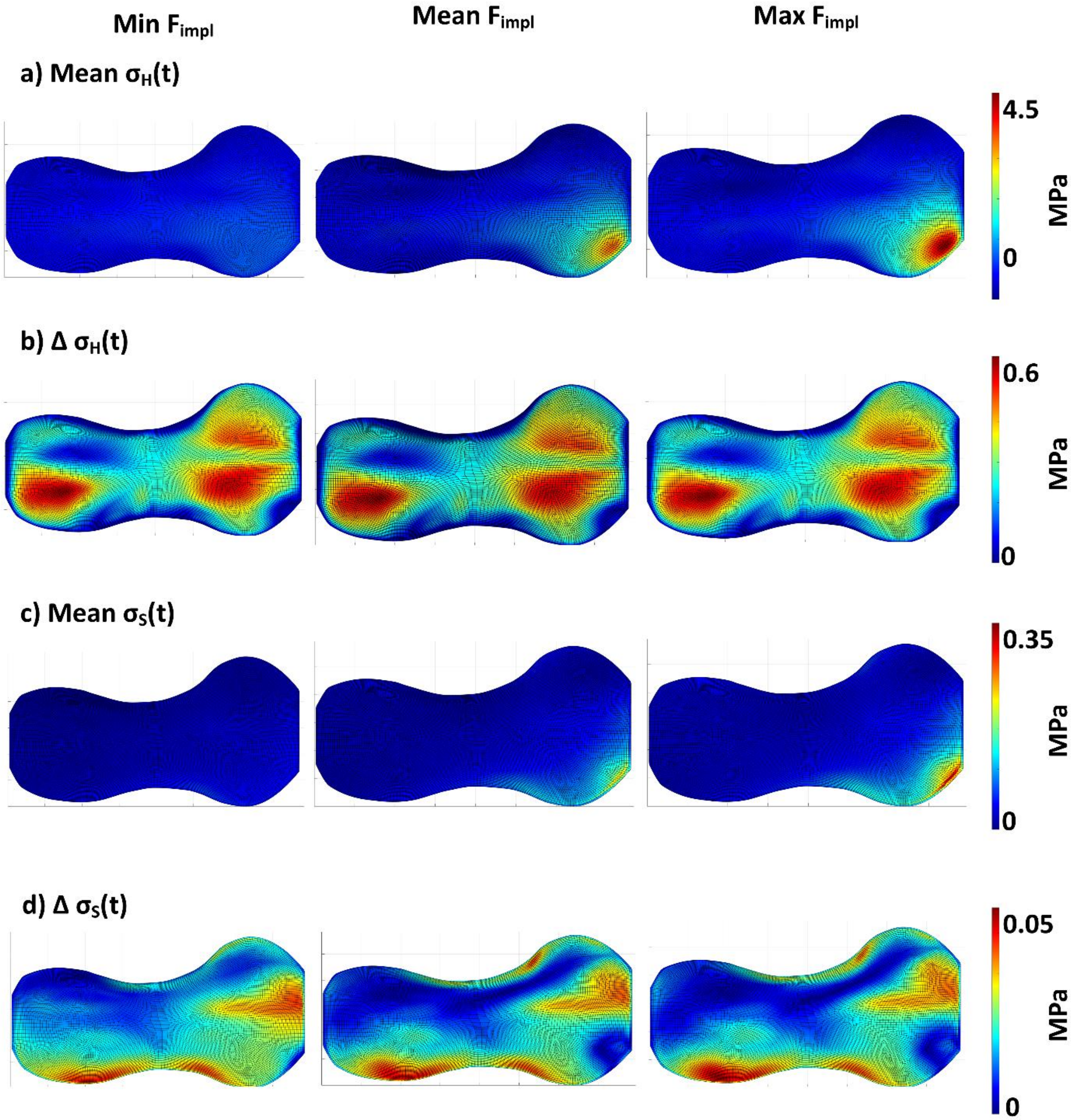

Supplementary Figure 26: Results of the minimum, mean and maximum implant force as boundary conditions on model M3 of patient **P2 R**. Sorted in Columns, the first column shows the minimum implant force (Min F_{impl}), the second shows the mean (Mean F_{impl}), and the third shows the maximum (Max F_{impl}). a) shows the results for the mean hydrostatic stresses over the gait cycle (Mean $\sigma_H(t)$). While at the minimum implant force there is little to no static pressure in the growth plate, with higher implant forces, the magnitude of the static pressure gets higher. The same applies to c) the mean octahedral shear stresses (Mean $\sigma_H(t)$). The peak-to-peak-amplitude over the gait cycle is not significantly influenced by the implant forces, as shown in b) for the hydrostatic stresses ($\Delta\ \sigma_H(t)$) and d) for the octahedral shear stresses ($\Delta\ \sigma_H(t)$).

P3 R

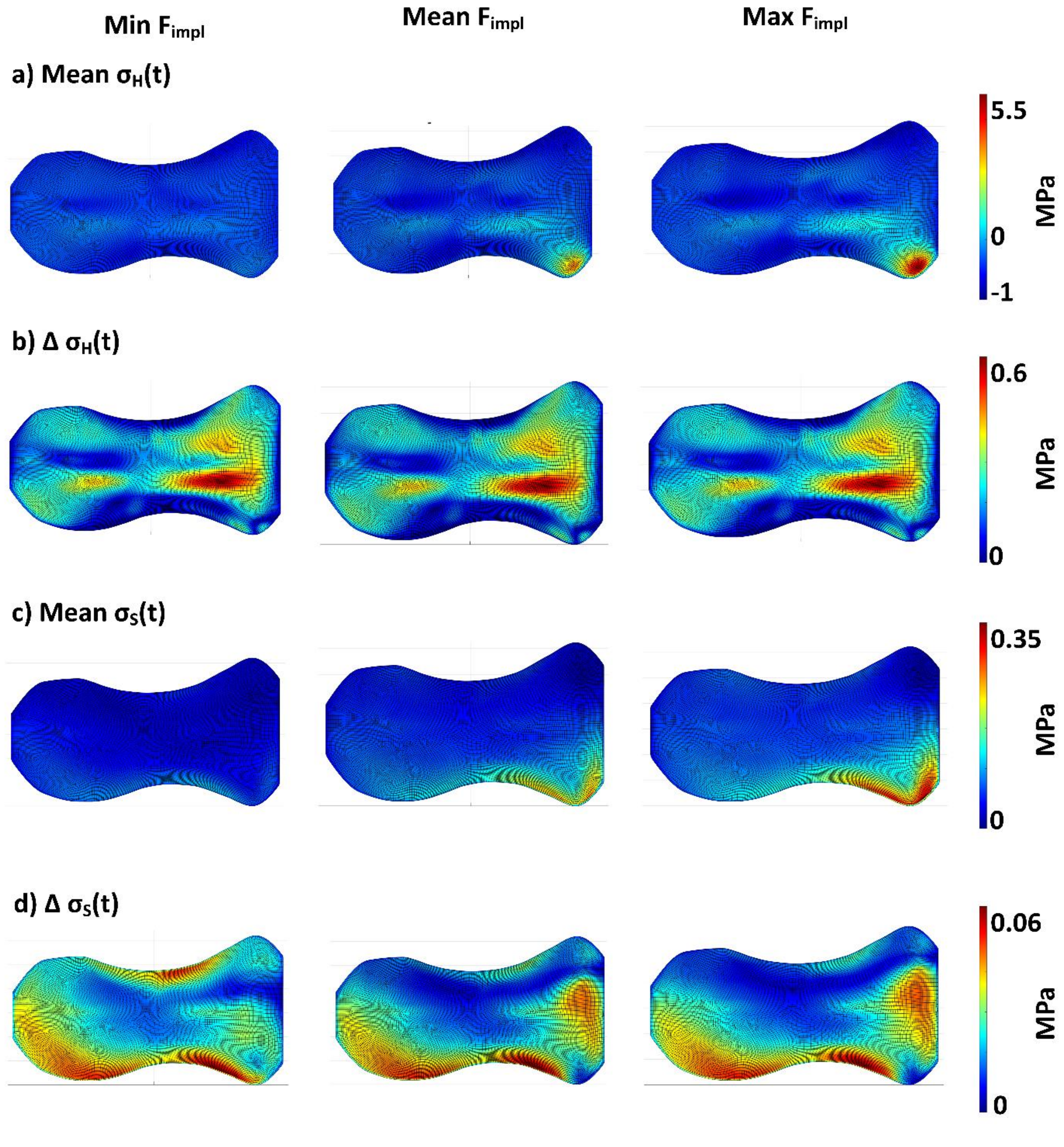

Supplementary Figure 27: Results of the minimum, mean and maximum implant force as boundary conditions on model M3 of patient **P3 R**. Sorted in Columns, the first column shows the minimum implant force (Min F_{impl}), the second shows the mean (Mean F_{impl}), and the third shows the maximum (Max F_{impl}). a) shows the results for the mean hydrostatic stresses over the gait cycle (Mean $\sigma_H(t)$). While at the minimum implant force there is little to no static pressure in the growth plate, with higher implant forces, the magnitude of the static pressure gets higher. The same applies to c) the mean octahedral shear stresses (Mean $\sigma_H(t)$). The peak-to-peak-amplitude over the gait cycle is not significantly influenced by the implant forces, as shown in b) for the hydrostatic stresses ($\Delta\ \sigma_H(t)$) and d) for the octahedral shear stresses ($\Delta\ \sigma_H(t)$).
